# Detection of *Xylella fastidiosa* in almond orchards by synergic use of an epidemic spread model and remotely sensed plant traits

**DOI:** 10.1016/j.rse.2021.112420

**Published:** 2021-07

**Authors:** C. Camino, R. Calderón, S. Parnell, H. Dierkes, Y. Chemin, M. Román-Écija, M. Montes-Borrego, B.B. Landa, J.A. Navas-Cortes, P.J. Zarco-Tejada, P.S.A. Beck

**Affiliations:** a European Commission (EC), Joint Research Centre (JRC), Ispra, Italy; b School of Environment and Life Sciences, University of Salford, Manchester, United Kingdom; c Instituto de Agricultura Sostenible (IAS), Consejo Superior de Investigaciones Científicas (CSIC), Cordoba, Spain; d School of Agriculture and Food, Faculty of Veterinary and Agricultural Sciences (FVAS), and Department of Infrastructure Engineering, Faculty of Engineering and Information Technology (FEIT), University of Melbourne, Melbourne, Victoria, Australia

**Keywords:** Hyperspectral, Thermal, Epidemic spread model, Radiative transfer model, SWIR domain, *Xylella fastidiosa*, Nitrogen, Machine learning

## Abstract

The early detection of *Xylella fastidiosa* (*Xf*) infections is critical to the management of this dangerous plan pathogen across the world. Recent studies with remote sensing (RS) sensors at different scales have shown that *Xf*-infected olive trees have distinct spectral features in the visible and infrared regions (VNIR). However, further work is needed to integrate remote sensing in the management of plant disease epidemics. Here, we research how the spectral changes picked up by different sets of RS plant traits (i.e., pigments, structural or leaf protein content), can help capture the spatial dynamics of *Xf* spread. We coupled a spatial spread model with the probability of *Xf-*infection predicted by a RS-driven support vector machine (RS-SVM) model. Furthermore, we analyzed which RS plant traits contribute most to the output of the prediction models. For that, in almond orchards affected by *Xf* (*n* = 1426 trees), we conducted a field campaign simultaneously with an airborne campaign to collect high-resolution thermal images and hyperspectral images in the visible-near-infrared (VNIR, 400–850 nm) and short-wave infrared regions (SWIR, 950–1700 nm). The best performing RS-SVM model (OA = 75%; kappa = 0.50) included as predictors leaf protein content, nitrogen indices (NIs), fluorescence and a thermal indicator (T_c_), alongside pigments and structural parameters. Leaf protein content together with NIs contributed 28% to the explanatory power of the model, followed by chlorophyll (22%), structural parameters (LAI and LIDF_a_), and chlorophyll indicators of photosynthetic efficiency. Coupling the RS model with an epidemic spread model increased the accuracy (OA = 80%; kappa = 0.48). In the almond trees where the presence of *Xf* was assayed by qPCR (*n* = 318 trees), the combined RS-spread model yielded an OA of 71% and kappa = 0.33, which is higher than the RS-only model and visual inspections (both OA = 64–65% and kappa = 0.26–31). Our work demonstrates how combining spatial epidemiological models and remote sensing can lead to highly accurate predictions of plant disease spatial distribution.

## Highlights

• RS models for *Xf* detection increase accuracy with pigments and leaf protein content.
• Anthocyanins increased with *Xf*-symptoms, while chlorophylls and N-cont. decreased.
• The coupled PSNFT-spread model increased the *Xf* detection, compared to RS-models.
• The predictions of a coupled RS-spread model yielded accurate *Xf* distribution maps.
• Coupled RS-spread model provides a novel tool for monitoring plant pests.

## 1 Introduction

The bacterium *Xylella fastidiosa* (*Xf*), described by Wells et al. (1987), is a xylem-limited gram-negative bacterial plant pathogen. *Xf* (accepted subspecies: *fastidiosa*, *pauca*, *multiplex*, *sandyi* and others, e.g., *morus*) is one of the most dangerous bacteria worldwide for plants. It affects over 30 families of monocotyledons and dicotyledons (Sherald and Kostka, 1992) and leads to environmental, social and economic damages. *Xf*-caused diseases are considered a global threat and recent outbreaks in Europe and Israel have impacted locally cultivated plants of high economic value (e.g., olive, plum, almond and cherry trees) as well as ornamental plants (e.g., myrtle-leaf milkwort, oleander). The emergence of *Xf* in new areas and the unsuccessful containment of its spread in territories where it is already established highlight the need to monitor the progress of large *Xf* outbreaks and develop comprehensive pest management strategies. In this regard, remote sensing (RS) methods that respond to the plant traits most sensitive to *Xf* infection (e.g., Zarco-Tejada et al., 2018) could help to monitor and mitigate the progress of large *Xf* outbreaks across a range of crops.

Remote sensing methods to detect plant pests have greatly advanced in recent years (Herrmann et al., 2018; Abdulridha et al., 2019; Poblete et al., 2020). Yet, such methods still only rarely take into account the nutritional status of the plant host. Both nitrogen deficiency and many pathogen infections reduce chlorophyll pigment content (Tartachnyk et al., 2006) and consequently photosynthetic activity. Monitoring the nutritional, physiological and water status of plants susceptible to a particular pathogen can thus help detect when infection occurs. In recent years, remote sensing has made significant progress in developing methods for the quantification of nitrogen content using the NIR and short-wave infrared (SWIR) spectral regions (Herrmann et al., 2010; Gnyp et al., 2014; Mahajan et al., 2014; Pimstein et al., 2011; Camino et al., 2018a). In parallel, advances in thermography show the feasibility of using thermal infrared remote sensing to monitor water status and transpiration across an entire crop field (Meron et al., 2010; Gonzalez-Dugo et al., 2015). In the last years, sun-induced chlorophyll fluorescence (SIF) has received increasing attention for the monitoring of photosynthetic activity at global (Joiner et al., 2011; Koffi et al., 2015; Norton et al., 2017; Frankenberg and Berry, 2018) and local scales (Pérez-Priego et al., 2005; Daumard et al., 2012; Zarco-Tejada et al., 2016; Camino et al., 2019). Most of the existing approaches for retrieving SIF – whether at ground level or from airborne or satellite sensors – rely on the Fraunhofer Line Discrimination (FLD) principle (Plascyk and Gabriel, 1975) by combining solar irradiance and radiance emitted by the vegetation through the use of atmospheric O_2_ absorption features (Damm et al., 2015; Meroni et al., 2009; Moya et al., 2004). In this regard, recent work (e.g., Sabater et al., 2018; Cendrero-Mateo et al., 2019; Pacheco-Labrador et al., 2019) highlighted the requirements for fine spectral resolutions (e.g., ≤ 1 nm) to successfully retrieve SIF in absolute terms using the main atmospheric oxygen absorption lines. Nevertheless, sensors with coarser spectral resolution have been successfully used to estimate SIF through adequate strategies for assessing its relative variability in the context of stress detection (Damm et al., 2014; Zarco-Tejada et al., 2012, Zarco-Tejada et al., 2016, Zarco-Tejada et al., 2018) and demonstrated theoretically by radiative transfer modelling (Damm et al., 2011).

Vegetation indices are commonly designed based on narrow-band reflectance to maximize sensitivity to vegetation characteristics such as chlorophylls pigments (Haboudane et al., 2002; Zarco-Tejada et al., 2004; Yu et al., 2015; Wang et al., 2017), carotenoids (Gitelson et al., 2002; Zarco-Tejada et al., 2013b), anthocyanins (Gitelson et al., 2006) and macronutrients (Herrmann et al., 2010; Pimstein et al., 2011; Mahajan et al., 2016; Camino et al., 2018a). However, these empirical approaches require appropriate modelling strategies to mitigate the structural and shadow effects on canopy reflectance. This can be partly overcome by using physical radiative transfer models (RTMs), which offer greater robustness and transferability (Jacquemoud and Baret, 1990; Zarco-Tejada et al., 2004; Schlerf and Atzberger, 2006; Wang et al., 2015). Several inversion methods for RTMs exist to retrieve leaf biochemical and biophysical plant traits at canopy scales. In fact, one of the most widely used RTMs is the PROSPECT leaf model (Jacquemoud and Baret, 1990) or its most recent version PROSPECT-PRO (Féret et al., 2020), which enable the quantification of leaf protein content. This model can be coupled to homogeneous approximations of canopies, such as SAIL (Verhoef, 1984) or used for complex canopy assumptions with 3-D simulations such as FLIGHT (North, 1996) or DART (Gastellu-Etchegorry et al., 1996).

In recent years, RTM inversion methods have been successfully improved using so-called hybrid methods (Berger et al., 2020; Brede et al., 2020; De Grave et al., 2020; Upreti et al., 2019). These hybrid approaches, described in detail by Verrelst et al. (2019), combine physically-based models with advanced non-parametric regression models, such as artificial neural networks, random forest (RF) regressions, support vector machines (SVM) or Gaussian process regression. As shown in   Verrelst et al. (2019), hybrid inversion methods have successfully been used to retrieve plant traits. Doktor et al. (2014), in particular, showed that RF approaches are able to retrieve LAI and chlorophyll content. Others developed techniques to estimate C_ab_ and structural parameters include the use of support vector machines (Liang et al., 2016), ensemble techniques that use RF-SVM regressions (Rivera-Caicedo et al., 2017) or the bagging trees methods (Upreti et al., 2019). Abdel-Rahman et al. (2013) showed the feasibility of RF regression and spectral band selection for estimating leaf nitrogen concentration. Berger et al. (2020) used a hybrid ML method with Gaussian regression to estimate crop nitrogen based on the coupled 4SAIL model with the leaf model PROSPECT-PRO.

Much remote sensing progress has been made using machine learning algorithms for detecting plant pests at the ground and orchard scales (Calderón et al., 2015; Herrmann et al., 2018; Zarco-Tejada et al., 2018; Abdulridha et al., 2019). However, most RS methods do not consider a priori information on the pathogen's spatial distribution, host landscape connectivity or epidemiological processes, such as pathogen dispersal mechanisms. However, disease spread models increasingly underpin disease control interventions and make them more targeted. Recent studies by Calderón and Parnell (2019) have shown how combining remote sensing-based plant health estimates with a stochastic disease spread model overcomes the limitations of using each approach separately. The disease spread model captured the impact of landscape connectivity and spatial disease dynamics building on previous research by Parnell et al., 2011, Parnell et al., 2017. It resulted in accurate maps of the spatial correlations typical of vector-borne transmitted diseases and quantified the effect of host spatial structure and landscape connectivity on disease distribution in olive trees infected by *Xf* subsp. *pauca* in the south of Italy. This combination of remote sensing and modelling approaches improved the detection rate of pre-visual *Xf* infections. Given that infected but asymptomatic trees contribute as an inoculum to the spread of *Xf* epidemics, such detections of pre-visual infections are critical to eradicate the disease.

Here, our aim was to develop a remote sensing method to map the spatial dynamics of *Xf* outbreaks and, specifically, to optimize the early detection of *Xf* infection in almond trees. To do that, we developed a remote sensing-based classification model that relied on a support vector machine (RS-SVM) and was driven by plant traits inverted with biophysical models (i.e., leaf nitrogen content, pigments, structural parameters) and a set of spectral indices. We then coupled the remote sensing model with an iterative stochastic spread model to estimate the spatial distribution of *Xf* in almond orchards. Finally, we assessed which of the predictor variables in the RS-SVM classification models were most critical to quantifying the *Xf* symptoms observed across the different almond orchards.

## 2 Material and methods

### 2.1 Field data collection

Incidence and severity of *Xf* symptoms were visually assessed by plant pathologists in 1426 almond trees distributed over 20 orchards naturally infected by *Xf* (subsp. *multiplex)*, in 9 municipalities of Alicante province, Spain. The assessment was carried out between 7 and 11 July 2018. *Xf* disease severity (DS) assessments consisted of visual inspection of *Xf* foliar symptoms, rating each almond tree on a 0–4 scale based on the fraction of the crown canopy with disease symptoms (DS), where zero corresponds to no visual symptoms (i.e., asymptomatic), one, two and three correspond to trees with visual *Xf* symptoms in between 1 and 25%, 25–50% and 50–75% of the tree-crown, respectively, and four corresponds to a tree with mostly dead branches (≥75% of the crown canopy; with leaf collapse or leaf scorch). Of the inspected trees, 46% were asymptomatic (DS_0_) and 54% showed *Xf* disease symptoms (sample sizes: n_DS0_ = 657, n_DS1_ = 359, n_DS2_ = 214, n_DS3_ = 142, n_DS4_ = 54).

Quantitative Polymerase Chain Reaction assays (qPCR) confirmed the presence of *Xylella fastidiosa* (subsp. *multiplex)* in all the almond orchards studied. Of the visually assessed trees, 318 (22%; of which 78% positive and 22% negative) – comprising both *Xf*-symptomatic and asymptomatic trees – were randomly selected for qPCR assays from each of the 20 orchards. Mature branches and cuttings with attached mature leaves were sampled following the standard protocol of the European and Mediterranean Plant Protection Organization (EPPO) for *Xylella fastidiosa*: PM 7/24(3). DNA extraction was performed using CTAB buffer from 0.5 g of xylem tissue samples. All samples were subjected to two qPCR assays using the real-time PCR tests of Harper et al. (2010, erratum 2013) and (Francis et al., 2006).

In a subset of all 1426 almond trees (*n* = 90) randomly selected for qPCR testing regardless of DS, we took leaf physiological measurements, in the central areas of the leaves, during the field campaign. Steady-state leaf chlorophyll fluorescence (Ft) was measured in 15/25 asymptomatic/symptomatic leaves per tree using the FluorPen FP-100 device (Photon Systems Instruments, Brno, Czech Republic). In total Ft was measured on 1135 asymptomatic leaves and 752 *Xf*-symptomatic leaves. For stomatal conductance measurements, we used a steady-state diffusion porometer (model SC-1, Decagon Devices, Washington, DC, USA). Stomatal conductance measurements, which are highly time-consuming, were only taken in 2/3 asymptomatic/symptomatic leaves in 10 trees located in the first almond orchard studied. Leaf reflectance – within the visible and near-infrared domains (400–800 nm) – was measured in 7/10 asymptomatic/symptomatic leaves per tree using the PolyPen RP400 handheld spectrometer (Photon Systems Instruments, Brno, Czech Republic). In total we measured leaf reflectance in 697 asymptomatic leaves and 417 symptomatic leaves. In the same trees and leaves, chlorophyll content (C_ab_), anthocyanin content (A_nth_), flavonoid concentration, the nitrogen balance index (NBI) and leaf temperature were measured in 15/25 asymptomatic/symptomatic leaves (Table 1; *n* = 2534 leaf samples) per tree using a leaf-clip Dualex 4 sensor (Force-A, Orsay, France). The relationship between chlorophyll content and Dualex readings for dicotyledon crops found by Cerovic et al. (2012) was applied to compute leaf chlorophyll content (μgcm^−2^) from the Dualex readings. We combined the Dualex readings measured in asymptomatic and *Xf-*symptomatic leaves of 90 almond trees with the information provided by the qPCR assays and visual inspections. After that, we classifed the Dualex readings into four categories based on the visual inspections and the qPCR tests (Table 1).

**Table 1:** Dualex readings classified into four categories based on *Xylella fastidiosa* disease severity (DS) according to the visual inspections and the qPCR test. A total of 2534 leaf-level measurements were used.

Category	Leaf sample	Visual inspection	qPCR tests	*n*
Healthy	Asymptomatic	DS = 0	Negative	372
Asymptomatic *Xf*-infected	Asymptomatic	DS ≥ 1	Positive	1113
Symptomatic *Xf*-infected	*Xf*-symptomatic	DS ≥ 1	Positive	1016
Non-*Xf*-infected	Asympt*/Xf*-sympt.	DS ≥ 1	Negative	33

We assessed the effects of *Xf* infection on stomatal conductance and leaf fluorescence using ANOVA analysis. For Dualex readings, prior to the analyses, we tested the data for normality using the Shapiro-Wilk test and for homogeneity of variances using the Kruskal-Wallis test; results of both tests were significant (*P* < 0.05). Consequently, we performed a Kruskal-Wallis test to assess the effects of *Xf*-infection status on Dualex parameters (C_ab_, A_nth_ and NBI) followed by the Wilcoxon post-hoc test with Bonferroni correction to estimate differences (*P* < 0.05) between the four classes shown in Table 1 (Healthy, Asymptomatic *Xf-*infected, Symptomatic *Xf*-infected and Non-*Xf*-infected).

To analyze spectral and pigment changes according to *Xf* symptoms, we measured leaf reflectance and took Dualex readings in asymptomatic and *Xf*-symptomatic leaves with different levels of *Xf*-symptoms (supplementary Fig. 1). To analyses the *Xf*-symptoms in this leaf dataset, we randomly measured 7/10 leaf measurements in different areas of the sampled leaves (5–6 leaves per class). This dataset was exclusively used to assess how pigments and nitrogen proportions change in leaves as *Xf* symptoms increase.

### 2.2 Airborne campaign

**Fig. 1:**
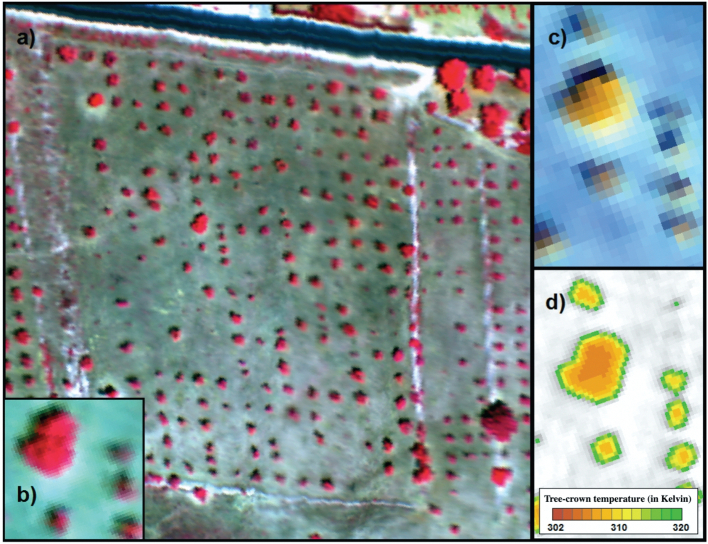
Overview of an almond orchard imaged by the hyperspectral VNIR sensor (a: composite: 800(R), 679 (G) and 540 (B) nm) during the airborne campaign conducted in Alicante in July 2018. A detailed view of the central almond trees is displayed in (b), (c, composite: 985 (R), 1285 (G) and 1550 (B) nm) and (d) using the hyperspectral VNIR, hyperspectral NIR/100 and the thermal sensor respectively. The spatial resolutions of hyperspectral and thermal sensors were 0.30 (a, b), 0.80 (c) and 0.40 (d) cm per pixel.

An airborne campaign was conducted on 10 July 2018 in Alicante province, central-eastern Spain (total flight area = 20,670 ha), using a manned aircraft with two high-resolution hyperspectral sensors (Fig. 1) and a thermal sensor installed in tandem: a VNIR micro-hyperspec imager (Headwall Photonics, Fitchburg, MA, USA), a micro-hyperspec NIR-100 linear-array imager (Headwall Photonics, Fitchburg, MA, USA) and the FLIR SC655 thermal camera (FLIR Systems, Wilsonville, OR, USA). To minimize differences due to sun angle effects, the flights were performed around noon (11–14 local time, range of solar zenith angle: 15–30 degrees) in clear-sky conditions. The aircraft flew on the solar plane at 250–400 m above ground level (AGL) and a cruising speed of 75–110 knots. The configuration of hyperspectral sensors used during the airborne campaign is summarized in supplementary Table S1.

The hyperspectral sensors were radiometrically calibrated in the laboratory with the CSTM-USS-2000C integrating sphere (LabSphere, North Sutton, NH, USA) at four levels of illumination using six integration times. The full width at half maximum (FWHM) was derived after spectral calibration using a Cornerstone 260 1/4 m Monochromator (model 74,100; Oriel Instruments, USA) and the XE-1 Xenon Calibration Light Source (Oceanic Optics, USA). Hyperspectral imagery was atmospherically corrected using the solar incoming irradiance (E) measured in the field concurrently with the flights through a handheld field spectrometer (FieldSpec Handheld Pro, ASD Inc., Longmont, Colorado, USA) with 3 nm bandwidth and a cosine corrector-diffuser probe for the VNIR sensor and simulated by the SMARTS model (Gueymard, 1995, Gueymard, 2001) for the NIR-100 sensor. To simulate incoming irradiance, aerosol optical measurements (air mass, aerosol optical depth at 550 nm and Ångström exponent at 550 nm) and simultaneous readings of relative humidity, temperature and air pressure were acquired at flight time with a Microtops II handheld multichannel sun photometer (Solar Light, Philadelphia, USA) connected to a GPS-12 model (Garmin, Olathe, KS) and a portable weather station (Transmitter PTU30, Vaisala, Helsinki, Finland), respectively. In addition, reflectance and radiance measurements were acquired at the flight time over three different surfaces (black, white and soil targets) using a handheld field spectrometer calibrated with a Spectralon white reference panel (SRT-99-180, LabSphere, NH, USA). The non-uniform illumination effects were corrected through a cross-track illumination correction. The irradiance was resampled through a Gaussian convolution to the bandwidth of each sensor. A further step was taken to correct the hyperspectral signal with the field spectrometer through an empirical line calibration (Smith and Milton, 1999) using the soil reflectance measured in the soil targets. To reduce noise in both hyperspectral sensors, we applied a Savitzky–Golay filter (Savitzky and Golay, 1964) to the reflectance signal.

The thermal camera on the aircraft had a resolution of 640 × 480 pixels with 16-bit radiometric resolution and 13.1 mm focal length, providing an FOV of 45 × 33.7°. This yielded a ground resolution of 40 cm during this flight (Fig. 1c). The radiometric calibration was performed using a blackbody (model P80P, Land Instruments, Dronfield, UK) and vicarious calibration performed for the flight using temperature measurements during the flight over black, white and soil targets with a handheld infrared thermometer (LaserSight Optris, Berlin, Germany). After each flight, thermal imagery was processed in the laboratory and mosaicked using Pix4D software to generate the entire scene.

### 2.3 Fluorescence emission, thermal indicator and plant stress-related vegetation indices

Average tree-crown radiance (L) and reflectance (R) spectra and tree-crown temperature (T_c_) were extracted from sunlit pure vegetation pixels using automated object-based methods. As a first step, the soil background and shadow effects within almond tree crowns were removed using object-based tree-crown segmentation. The tree crowns were segmented using Niblack's thresholding method (Niblack, 1986) and Sauvola and Pietikäinen's binarization techniques (Sauvola and Pietikäinen, 2000). As a second step, a watershed segmentation based on the Euclidean distance was applied to separate touching or overlapping tree crowns in the images. Radiance spectra in sunlit pure vegetation pixels were used to quantify the solar-induced chlorophyll fluorescence (SIF) signal using the FLD in-filling method (Plascyk and Gabriel, 1975), which was well described by Moya et al. (2004) and Meroni et al. (2010). This step is important when attempting to estimate SIF with coarser spectral resolution sensors (5–6 nm), mainly because the fluorescence signal retrieved from mixed pixels (e.g., branches, shadows) is strongly affected by illumination effects and canopy structure (Camino et al., 2018b; Aasen et al., 2019) causing an overestimation of the fluorescence quantification. The FLD method is based on the SIF retrieval using two spectral bands located in and out of the O_2_–A absorption feature located at 760.5 nm (Fig. 5a). Despite the high spectral resolution (< 1 nm) needed to quantify SIF at the O_2_-A, modelling work by Damm et al. (2011) demonstrated that sensors with 5–6 nm bandwidth within the O_2_ absorption window can be used to derive chlorophyll emission using the FLD method, which is typically estimated using instruments with a spectral resolution below 0.5 nm. In particular, The FLD_2_ method used L_in_ (L_762_ nm) and L_out_ (L_750_ nm) from the hyperspectral VNIR sensor, and solar incoming irradiance E_in_ (L_762_ nm) and E_out_ (L_750_ nm) concurrently measured at the time of the flight with the field spectrometer. The average reflectance (Fig. 4b) extracted from pure vegetation pixels in single tree crowns was used to calculate several commonly used plant-stress spectral indices found in the scientific literature. More than 90 narrow spectral indices were calculated using 420 spectral bands located in the visible-NIR and the short-wave infrared regions. These indicators were related to pigment content, structure, epoxidation state of the xanthophyll cycle, chlorophyll fluorescence emission, blue-green-red ratio indices and nitrogen indices (NIs) using the SWIR domain. To reduce the number of narrow spectral indices, we conducted a multicollinearity analysis using the variance inflation factor (VIF) and Wilks' lambda scores to identify the non-correlated narrow spectral bands that made the most significant contribution to early detection of *Xf* in almond trees (supplementary Table S2). The analysis of the variance factor of inflation (VIF) was calculated for each narrow-band index by linear regression with the other narrow-band indices. After that, the VIF of that regression was obtained using the equation VIF = 1/(1-*r*^*2*^; where *r*^*2*^ is the coefficient of determination). To reduce the high inter-correlations or inter-associations among the narrow-band indices, a VIF threshold ≤10 was chosen (Hair et al., 1995). Subsequently, a stepwise forward variable selection using Wilks' Lambda criterion with an approximate F-test decision ≤0.15 was performed to select the most significant plant traits to distinguish between asymptomatic tree crowns (DS = 0) and tree crowns with *Xf* symptoms (DS ≥ 1). After that, as the spectral data were not normally distributed at orchard level, the Kruskal-Wallis test and the Wilcoxon post-hoc test with Bonferroni correction were performed to evaluate the significant differences in the main spectral indices between asymptomatic tree crowns (DS = 0) and tree crowns with *Xf* symptoms (DS ≥ 1).

### 2.4 Inversion of plant traits using an RTM-RF hybrid approach

We retrieved the canopy structural parameters and leaf biophysical and biochemical constituents from each tree crown by PROSAIL-PRO inversion using the average reflectance spectra extracted from pure-vegetation pixels based on similar tree-crown segmentation approaches taken by Zarco-Tejada et al. (2018) and Poblete et al. (2020). The PROSAIL-PRO radiative transfer model couples the PROSPECT-PRO leaf reflectance model (Féret et al., 2021), with the 4SAIL turbid medium canopy radiative transfer model (Verhoef et al., 2007). PROSPECT-PRO model enabled the separation of the nitrogen-based constituents (proteins) from  carbon-based constituents (including cellulose, lignin, hemicellulose, starch and sugars). The SAIL model is based on the 1-D model developed by Suits (1971) to simulate the bidirectional reflectance of a canopy.

An RTM-based hybrid inversion with random forest regression was used to estimate each biochemical and biophysical parameter using the reflectance spectra from the RTM simulations and from the hyperspectral sensors in the 400–1750 nm spectral domain. To avoid overfitting on the training sample, a 10-fold cross-validation with 5 repeats was used in the RF models to predict each plant trait. The RF machine learning approach was based on 40,000 simulations using a look-up table (LUT) with the biochemical and biophysical ranges shown on Table 2. A uniform distribution was selected for each varied plant parameter. The solar geometry – defined by the solar zenith and azimuth – and the viewing angles needed to simulate canopy reflectance were extracted for the flight date. The solar zenith angle was set between 0 and 45 degrees to minimize the impact of the viewing geometry at each flight date and time. To avoid potential ill-posed inversion solutions during the inversion method, we constrained all inputs in the LUT based on field measurements, existing literature, and preliminary simulations to make sure that the resulting LUT covered range of the observations made by the hyperspectral sensors over the tree crowns.

**Table 2:** Input variables used in the coupled PROSAIL-PRO model.

Input	Parameter	Ranges	Units
PROSPECT-PRO			
N	Leaf structure parameter	1.5–2.5	[−]
C_ab_	Chlorophyll a + b content	0–60	μg/cm^2^
C_ar_	Carotenoid content	0–15	μg/cm^2^
A_nth_	Anthocyanin content	0–7.5	μg/cm^2^
C_brown_	Brown pigment	0–1	[−]
C_w_	Equivalent water thickness	0.004–0.012	g cm^-2^
C_p_	Leaf protein content	0.0004–0.005	g/cm^2^
CBC	Carbon-based constituents	0.001–0.002	g/cm^2^
SAILH 5B			
LAI	Leaf area index	0.5–4	m^2^ m^-2^
LIDF_a_	Leaf angle distribution (a)	30–90	Deg
LIDF_b_	Leaf angle distribution (b)	1	[−]
hspot	Hotspot parameter	0–1	Deg
Tts	Solar zenith angle	0–45	Deg
Tto	Observer zenith angle	0–25	Deg
Psi	Relative azimuth angle	0	Deg
R_soil_	Soil reflectance	From image	[−]

In order to remove data noise affecting model inversion, we applied a smoothing of the simulated spectrum at 1 nm resolution using a Savitzky-Golay derived calculation method (Savitzky and Golay, 1964). After that, we resampled each simulated spectrum to adjust its resolution to the bandwidth of the sensors using gaussian spectral response functions defined by the FWHM values of each sensor (FWHM = 6.5 nm for the VNIR hyperspectral sensor and FWHM = 6.05 nm for the NIR-100 hyperspectral sensor).

Next, we trained a set of RF regression models to estimate plan traits from synthetic canopy reflectance data to obtain individual plant traits. Resampled reflectance simulations at hyperspectral resolution were partitioned using a random selection into two groups: the training sample with 75% of the simulations and the testing sample with the remaining 25% of simulations. After that, the performance of each RF model for retrieving individual plant traits was evaluated by calculating the coefficient of determination (r^2^), the root mean square error (RMSE) and the mean absolute error (MAE).

Finally, we applied the RF regression models to the airborne hyperspectral reflectance to estimate plant traits for individual tree crowns. Performance for the inversions through PROSAIL-PRO are listed supplementary Table S3. We evaluated the uncertainties of the chlorophyll and anthocyanin concentrations retrieved at tree crown level with leaf-level Dualex measurements (supplementary Fig. S2 and S3). For that, we assessed the inverted C_ab_ and A_nth_ with the average leaf Dualex readings grouped by leaf-level disease severity level (supplementary Fig. S2 and S3). In the absence of field measurements, the structural parameters (LAI and LIDFa) could only be compared with related narrow band indices (i.e., NDVI and SIF, supplementary Fig. S4). Additionally, we used correlation maps based on the normalized difference spectral index (NDSI) to analyze the sensitivity of each plant trait (LAI, LIDF_a_, C_w_ and C_p_) to all possible band combinations in the 400–1700 nm region (supplementary Fig. S5). Finally, to explore how well individual plant traits could distinguish asymptomatic almond trees from those with initial or advanced *Xf* symptoms, we compared all traits to the DS reported during the visual inspection (supplementary Fig. S6-S8). The statistical analyses were conducted with R software (R Core Team, 2020); the spectral transformation was performed with the ‘hsdar’ R package (Lehnert et al., 2019) and the RFs were implemented with the ‘caret’ package (Kuhn, 2020).

### 2.5 Machine learning classification to detect *Xylella fastidiosa* infection

We used non-linear support vector machines (SVMs) to classify disease incidence in almond tree crowns, using the visual assessments as reference (*n* = 1426 trees) and the RS plant traits as predictors. The SVM algorithm is based on statistical learning theory and estimates the hyperplane that optimally separates between classes. The non-linear SVM classification method was applied using the radial basis function kernel and a cost function to penalize errors associated with the misclassification of tree crowns. The best radial function and cost parameter were found using a grid search method with a cross-validation approach.

The remote sensing-based SVMs were applied to assess the separation between asymptomatic tree crowns (DS = 0) and *Xf*-symptomatic tree crowns (DS > 0). First, the set of plant trait predictor variables, estimated from the remotely sensed images, was reduced by a VIF analysis with a threshold ≤10 and Wilks' lambda method with an F-test decision ≤0.15. The VIF- Wilks' lambda approach allowed us to obtain the most parsimonious model with the least number of plant traits for each VNIR and SWIR spectral regions. Next, we built the SVM models using three different sets of functional plant traits: i) the pigment- and structure-based functional traits (PS) composed of selected VIF-Wilks' lambda indices in the VNIR and plant traits retrieved by RTM inversions (C_ab_, A_nth_, C_ar_, C_w_, LAI, and LIDF_a_); ii) the pigment-structure-based, leaf protein content (C_p_) and nutrition-based traits selected by the VIF-Wilks' lambda in the NIR-SWIR (PSN); and iii) the pigment-structure-nutrition-based traits, fluorescence and thermal-based functional traits (PSNFT), which also include the chlorophyll fluorescence emission (described in Section 2.3) and crown temperature (T_c_) extracted from the thermal imagery. In addition, we evaluated the effect of fluorescence, temperature, and C_p_ individually on the overall accuracy of the PS model. This is important from an operational point of view, in order to weigh the costs and benefits of installing additional high-resolution sensors on aerial platform to detect the *Xf* outbreaks.

Each of the three SVM models (PS, PSN and PSNFT), was run over the data set in 80 iterations. Each iteration selected 75% of the trees for model training, reserving the remainder for testing and maintaining the 1:1 ratio between symptomatic and asymptomatic of the original dataset through balancing techniques. In addition, we included K-fold (10-fold) cross-validation to avoid overfitting problems. The classification accuracies of the different RS-SVM models were evaluated by calculating the overall accuracy (OA, in %), the kappa value (Cohe, 1960) and the Area-Under-the-Curve (AUC) scores. OA and Cohen's kappa are calculated based on the confusion matrix. The AUC is estimated through the receiver operating characteristic (ROC) curves using the prediction probabilities of each model and the disease incidence recorded during the visual inspections. Moreover, we conducted an importance analysis for each SVM (PS, PSN and PSNFT) to analyze the contribution of each plant trait in the RS-SVM models based on the weight of coefficients of each SVM model. Finally, we assessed the classification accuracy of the proposed remote sensing SVM disease detection model and the visual evaluation against the results of the qPCR assays in each orchard studied.

### 2.6 Integrating spread modelling and remote sensing to optimize the early detection of *Xf* infection

We coupled a spatially explicit stochastic epidemic spread model with the RS-based infection estimations obtained from the PSNFT model. The method uses an iterative stochastic optimization algorithm to estimate the spatial distribution of disease from a sample by explicitly simulating the individual distance-dependent spread processes between the pathogen and its host population (Parnell et al., 2011). Thus, the probability of disease for each single unsampled tree is estimated by iterating through unsampled individuals at random, updating their probability of disease and accepting only map-improving changes. The resulting probability of disease was integrated into the epidemic spread model to quantify the contribution of host connectivity to the pathogen spread throughout the landscape with graph and circuit theories. The hosts in the landscape, mapped using the high-resolution imagery, were interconnected to build a graph where each tree was connected to its eight nearest neighboring trees. Next, the probability of disease was allocated to each tree, using RS-based probability estimations in unsampled trees and disease status determined by visual inspection in sampled trees (i.e., 1 if the disease was present and 0 if the disease was absent). We tested the proposed method using multiple random sample placements with a range of sample sizes from 5 to 20% of all the inspected trees. Pathogen spread across the graph was modelled using circuit theory, where the effective distances (*deff*_*ij*_) between node pairs were used as proxies of effective pathogen dispersion (McRae et al., 2008). The effective distance between any pair of nodes was defined as the resistance distance between the nodes when each graph edge was replaced by a resistor, the conductance (*G*_*ij*_) of which means the dispersal probability between two individuals as follows:

(1) $$G_{\mathrm{ij}}=P_{i}\cdot \exp\left(-\beta \cdot d_{\mathrm{ij}}\right)\cdot \frac{P_{{\mathrm{edge}}_{\mathrm{ij}}}}{\sum_{j=1}^{8}P_{{\mathrm{edge}}_{\mathrm{ij}}}}$$

where *P*_*i*_ is the probability of disease of tree *i* estimated by RS; *P*_*edgeij*_ is the probability of pathogen dispersal across the edge *ij*, which was defined as the area of a rectangular trapezium where the length of the side perpendicular to the bases is equal to 1 and the bases were assigned *P*_*i*_ and *P*_*j*_, respectively; *d*_*ij*_ is the Euclidean distance in meters between tree *i* and tree *j*; and *β* is the distance decay coefficient (*β* > 0) of the negative exponential dispersal kernel, which had to be estimated. Based on this initial condition, we calculated the initial value of the objective function (OF), which was an accuracy metric of the estimated map. In this study, sampled trees refers to trees randomly selected from each orchard except for those where qPCR tests were performed to assess their *Xf*-infection status; this was the only information that was used to assess the accuracy of the estimated map. The deviance, which was based on likelihood statistics to make a comparison between binary observations and probability estimates (Parnell et al., 2011), was selected as the OF. The optimization process to minimize the OF made it possible to maximize the accuracy of the map. Subsequently, the iteration loop started by choosing an unsampled tree *i* at random and updating its probability of disease according to the function

(2) $$P_{i}=1-\exp\left[-\sum_{j\neq i}P_{j}\cdot \exp\left(-\alpha \cdot {\mathrm{deff}}_{\mathrm{ij}}\right)\right]$$

Here, *deff*_*ij*_ was the effective distance between unsampled tree *i* and (sampled or unsampled) tree *j*, where *α* was the distance decay coefficient (*α* > 0), which had to be estimated. Once this change was integrated into the system, the OF was recalculated using the eq. [2] the previous function to determine the probability of disease for each sampled tree by using the probability of disease of all the other trees (sampled and unsampled). If this new value of the OF was less than its value from the previous iteration, the change was accepted. Otherwise, it was rejected, and the previously estimated map and OF were retained. The stopping criterion was calculated from the gradient of the OF across the previous 1000 iterations of the algorithm. If this threshold gradient was <0.01, the algorithm was stopped and the current estimated map was considered as the final map. The selected combination of parameters was that with the lowest OF that led to the most accurate map. The estimated probabilities of disease were converted to binary presence/absence data using the average estimated probability across all unsampled trees as the threshold (Parnell et al., 2011). The coupled PSNFT-spread model was tested over the 20 orchards. To explore if the coupled RS-spread model actually improved the detection of the early symptoms at a pre-visual stage, we evaluated the accuracies of the PSNFT disease detection model, the coupled PSNFT-spread model, and the visual inspection using qPCR assay data obtained in the selected orchards.

## 3 Results

### 3.1 Spectral, pigment, nitrogen and physiological changes at leaf level

**Fig. 2:**
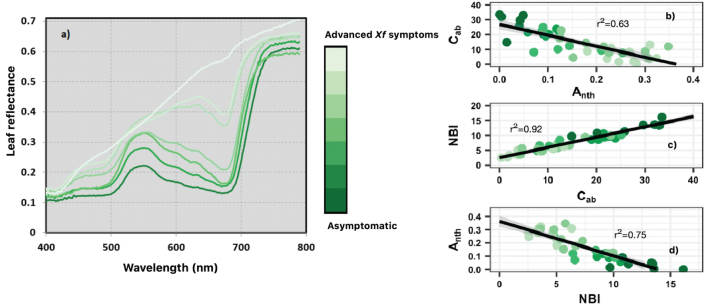
Leaf reflectance measurements (a) and scatterplots (b-d) between pigments (chlorophyll content (C_ab_) in μgcm^−2^ and anthocyanin content in Dualex units) and nitrogen balance index (NBI) collected from grouped leaves according to the severity level of leaf scorch symptoms caused by *Xylella fastidiosa* (*Xf*) (0–5 and leaf completely affected by *Xf* symptoms). The solid line shows the regression line.

Leaf reflectance and the Dualex readings were assessed in asymptomatic and *Xf*-symptomatic leaves. Leaf reflectance consistently increased with leaf DS level (Fig. 2a). The blue region (450–485 nm), the green region (495–570 nm), the red region (625–685 nm) and the red-edge peak (≃ 705 nm) showed the degradation of the chlorophyll pigments as leaf DS increased. In the blue and green regions, the reflectance profiles showed the degradation of the carotenoids as *Xf* symptoms worsened and spectral changes in the green region appeared linked to high anthocyanins levels (Fig. 2b). The scatterplots between pigments and NBI (Fig. 2b and d) showed that as the severity of *Xf* symptoms increased, anthocyanin levels also increased, while the chlorophyll and nitrogen content decreased.

**Fig. 3:**
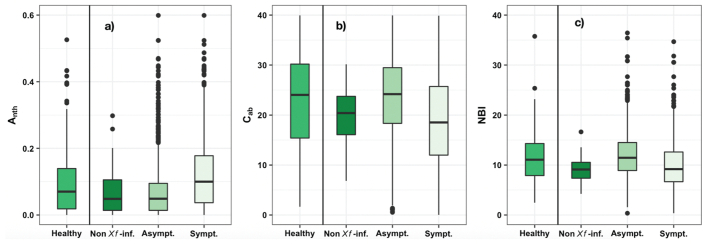
Dualex readings of anthocyanin (A_nth_; a), chlorophyll content (C_ab_ in μgcm^−2^; b) and nitrogen balance index (NBI; c) measured in almond leaves classified into four categories based on information from qPCR assays and visual inspections. *Healthy* refers to leaf-level measurements carried out on asymptomatic leaves where the absence of *Xf* bacteria was confirmed (qPCR = negative) and visual inspections assigned DS = 0; *Asympt*. refers to leaf-level measurements conducted on asymptomatic leaves in trees where the presence of *Xf* bacteria was confirmed by qPCR (positive) and DS ≥1; *Sympt.* refers to leaf-level measurements conducted on symptomatic leaves in trees where the presence of *Xf* bacteria was confirmed by both methods (qPCR = positive and DS ≥1); *Non-Xf-inf.* Refers to leaf-level measurements conducted on asympt./sympt. Leaves in trees where the visual inspection assigned DS ≥1, yet qPCR was negative.

When the Dualex readings were compared to *Xf*-infection status according to qPCR assays and visual inspection ratings, the Kruskal-Wallis test (Fig. 3; supplementary Table S4) showed significant differences (*P* < 0.05) for anthocyanins, chlorophyll and NBI between healthy leaves (qPCR = negative and DS = 0) and asymptomatic/symptomatic leaves measured in almond trees, where the presence of *Xf* bacteria was confirmed by qPCR tests (qPCR = positive and DS ≥1). However, it showed no significant differences (*P* ≥ 0.05) between symptomatic leaves sampled from non-*Xf*-infected trees (qPCR = negative and visual inspection DS ≥1) and *Xf*-symptomatic leaves. In general, anthocyanins exhibited lower values in healthy leaves (qPCR = negative and DS = 0) than in symptomatic *Xf*-infected leaves (qPCR = positive and DS ≥ 1) (Fig. 3a; supplementary Table S4). In fact, anthocyanin levels in healthy leaves were similar to those in asymptomatic leaves sampled from symptomatic *Xf*-infected trees (qPCR = positive and DS ≥1). However, chlorophyll and nitrogen values were inversely related to the occurrence of visible *Xf* symptoms (Fig. 3c; Fig. 3b and Fig. 2c).

**Fig. 4:**
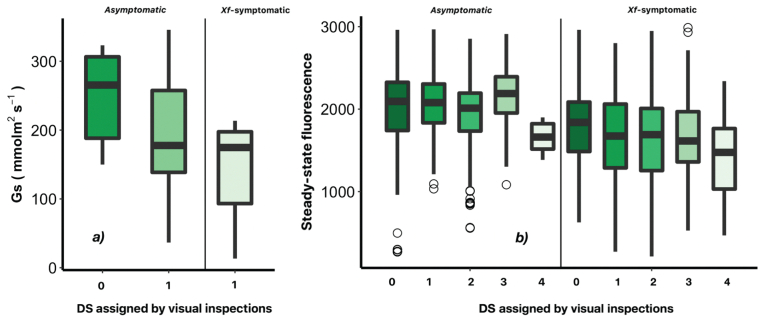
Stomatal conductance (Gs, in mmol.m^−2^.s^−1^) (a) and steady-state chlorophyll fluorescence emission (b, in arbitrary units) measured in asymptomatic leaves and symptomatic leaves affected by *Xylella fastidiosa*. The leaf samples were grouped by disease severity (DS) levels on a 0–4 rating scale assigned by the visual inspection of each sampled tree.

Leaf stomatal conductance (Gs) measured with the porometer in asymptomatic and *Xf*-symptomatic leaves in one orchard (Fig. 4a) showed that stomatal conductance decreased in *Xf-*infected leaves. Healthy asymptomatic leaves yielded maximum Gs values of 345 mmol.m^−2^.s^−1^, compared to 214 mmol.m^−2^.s^−1^ in leaves with *Xf* symptoms. The ANOVA analysis showed that stomatal conductance differed significantly (*P* < 0.05) between asymptomatic (DS = 0) and *Xf-*symptomatic leaves with DS ≥ 1.

Steady-state leaf chlorophyll fluorescence measured with the FluorPen FP-100 instrument (Fig. 4b) showed a downward trend as DS increased. *Xf-*symptomatic leaves showed lower Ft values than asymptomatic leaves. However, fluorescence is affected by the acquisition measurement time and diurnal factors, which varied across DS levels. Despite these diurnal differences, a clear pattern was observed between asymptomatic and symptomatic leaves in trees infected by *Xf*, reaching an average value of 2020 ± 426 arbitrary units (a.u.) and 1652 ± 544 a.u., respectively. The ANOVA confirmed statistically significant differences in Ft between asymptomatic and symptomatic leaves in trees at early and middle stages of *Xf* disease development (*P* ≤ 4.52 × 10^−9^ for DS 1–3).

### 3.2 Assessment of spectral indices and plant traits to track changes at tree-crown level

**Fig. 5:**
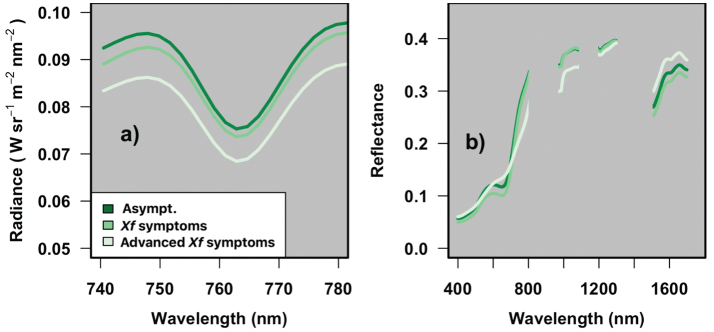
Average tree-crown radiance in Wsr^−1^ m^−2^ nm^−1^ (a) and mean tree-crown reflectance spectra in % (b) retrieved from hyperspectral imagery in tree crowns that were asymptomatic (DS = 0, n = 673 tree spectra), had *Xylella fastidiosa* (*Xf*) symptoms (1.5 <DS ≤ 2.5, n = 144 tree spectra) or had advanced *Xf* symptoms (DS ≥ 2.5, n = 55 tree spectra). To reduce noise in the radiance and reflectance spectra, the hyperspectral signal was convoluted using the Whittaker algorithms for this figure.

The average radiance spectra extracted from images of asymptomatic and *Xf*-affected trees (Fig. 5a) showed marked differences from the radiance used to estimate solar-induced chlorophyll emission. In particular, the FLD region showed higher radiance in asymptomatic trees (DS = 0, *n* = 673 tree-spectra) than in trees with *Xf* infection (1 ≤ DS ≤ 2.5, *n* = 144 tree-spectra) and advanced *Xf*-infected trees (DS ≥ 2.5, *n* = 55 tree spectra). This was consistent with the average reflectance spectra measured in pure vegetation pixels at tree-crown level (Fig. 5b), where asymptomatic trees showed lower reflectance in the VIS-NIR spectral region (400–1250 nm) than trees showing initial or advanced *Xf* symptoms. In the spectral range between 1500 and 1700 nm, the hyperspectral reflectance showed that asymptomatic trees had also lower reflectance than symptomatic trees with advanced DS levels.

**Fig. 6:**
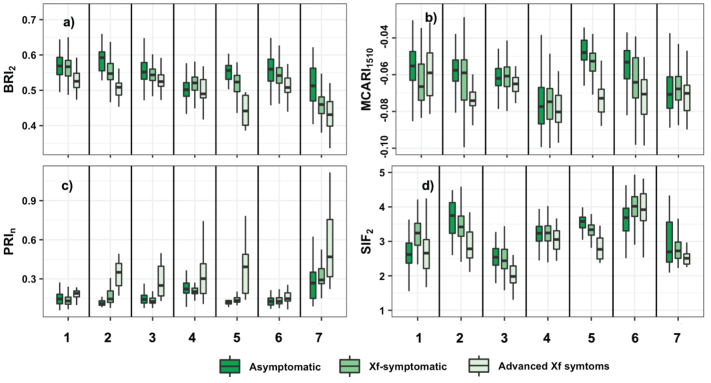
Blue reflectance index BRI_2_ (a), nutritional MCARI index at 1510 nm (b), PRI_n_ (c) and chlorophyll fluorescence emission (d) retrieved from hyperspectral imagery from tree crowns assessed as asymptomatic (DS = 0), with *Xylella fastidiosa* symptoms (1 ≤ DS ≤ 2.5), and with advanced *Xf* symptoms (DS ≥ 2.5) in almond orchards grouped by spatial location (seven groups labelled with numbers). (For interpretation of the references to colour in this figure legend, the reader is referred to the web version of this article.)

Based on the VIF analysis and Wilks' lambda, we found that the narrow-band spectral indices most sensitive to *Xf*-symptom were those related to chlorophyll pigments (i.e., TCARI, NPQI, VOG_2_, D_att_-CabC_x_ + c), the blue/green ratio (BF_1_), blue reflectance index (BRI_2_), photosynthetic efficiency (PRIn, PRI_M1_), chlorophyll fluorescence emission (SIF, CUR), and nitrogen content (MCARI_1510_, CI_2_, RSI, and GnyLi). We evaluated the main narrow-band indices and plant traits at the orchard level to evaluate whether they could track differences between asymptomatic and *Xf-*symptomatic trees under different conditions, such as almond varieties, canopy density (e.g., dense tree crowns vs. open tree crowns), flight time (e.g., irradiance levels) and soil spectral signals. In particular, the PRI_n_, showed a positive trend as *Xf* severity increased, while the MCARI at 1510 nm, BRI_2_ and SIF_2_ showed negative trends (Fig. 6). Overall, the Wilcoxon post-hoc (supplementary Table S5) test confirmed that the TCARI and PRI_n_ didn't differ significantly (*P* ≤ 0.005) between asymptomatic trees (DS = 0) and *Xf-*symptomatic trees in the majority of the orchards. The MCARI at 1510 nm and SIF_2_ did not show statistical differences between asymptomatic trees (DS = 0) and *Xf*-symptomatic trees for some orchards (Fig. 6d).

**Fig. 7:**
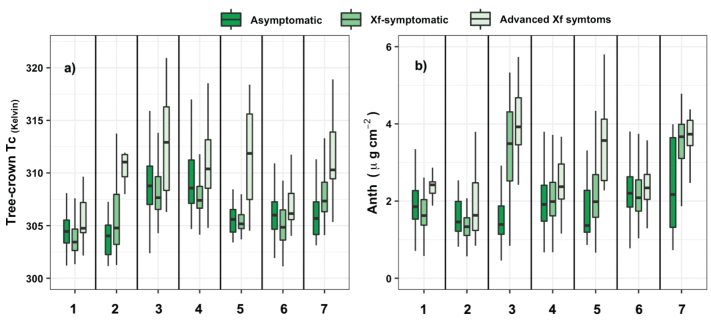
Tree-crown temperature (in Kelvin, a) and anthocyanin content (in μg/cm^2^, b) of tree crowns estimated using thermal imagery and RTM inversion, respectively, from tree crowns assessed as asymptomatic (DS = 0), with *Xylella fastidiosa* symptoms (1 ≤ DS ≤ 2.5) and with advanced *Xf* symptoms (DS ≥ 2.5) in almond orchards grouped by spatial location (seven groups labelled with numbers).

The crown temperature retrieved from pure vegetation pixels in the thermal imagery (Fig. 7a) trended similarly to the PRI_n_. Anthocyanin content also showed a positive trend DS levels increased (Fig. 7b). The Kruskal-Wallis and Wilcoxon post-hoc tests (supplementary Table S6) confirmed that crown temperature, leaf protein content, and anthocyanin content showed significant differences (*P* < 0.05) among asymptomatic, *Xf*-symptomatic and advanced *Xf*-symptomatic trees. The C_w_ and Car (not shown for sake of brevity) also captured the differences between asymptomatic trees and those with *Xf* symptoms. In contrast, the structural parameter LAI and chlorophyll content, in contrast, did not distinguish well between *Xf* symptomatic trees and those with advanced *Xf* symptoms in some plots.

### 3.3 RS-based SVM models and stochastic epidemic spread model for *Xf*-symptom detection

**Fig. 8:**
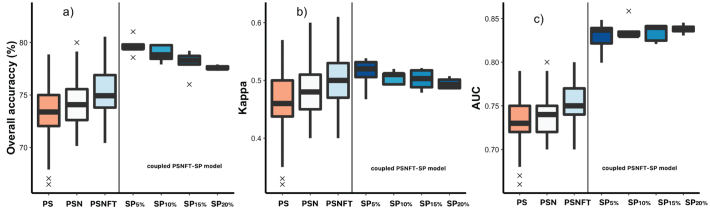
Overall accuracy (a), kappa (b) and area under the curve (AUC; c) for the RS-based SVM models (PS, PSN, PSNFT) and the coupled PSNFT-spread model. The RS-SVM models were tested with 25% of the 1426 almond trees in orchards affected by leaf scorch caused by *Xylella fastidiosa* using 80 random splits in the entire database. The coupled PSNFT-spread model (PSNFT-SP) was tested using multiple random sample placements at a range of sample sizes from 5 to 20% (step = 5).

We compared the accuracy of the statistical performances of the studied RS-based SVM models (i.e., PS, PSN and PSNFT) with the performance of the coupled PSNFT stochastic spread model using different sample sizes (5, 10, 15 and 20%). Fig. 8 shows the OA, in %), the κ, and the AUC scores estimated by the main non-linear SVM models and the coupled RS-PSNFT spread model for the testing samples. The PS model showed lower average accuracies (OA = 73 ± 2%, kappa = 0.46 ± 0.09 and AUC = 0.73 ± 0.04) than the PSN model (OA = 74 ± 2%, kappa = 0.48 ± 0.04 and AUC = 0.74 ± 0.02), which included four NIR/SWIR-based spectral indicators related to nitrogen status (RSI, CI_2_, GnyLi and MCARI_1510_) and leaf protein content. When the PSN model was combined with thermal-based T_c_ and solar-induced chlorophyll emission in the PSNFT model, performance increased to an average OA of 75 ± 2%, kappa = 0.50 ± 0.05 and AUC 0.75 ± 0.02 (supplementary Table S7). When the PSNFT model was coupled with the epidemic spread model, the overall average accuracy improved (OA = 80 ± 0.08%; kappa = 0.48 ± 0.02 and AUC = 0.81 ± 0.02) compared to that of RS-based SVM models (Fig. 8).

To assess the influence of random sample placement and sample size (5, 10, 15 and 20%), we estimated disease distribution maps in five simulations of the RS-spread model for each sample size. As expected, average AUC increased with sample size (AUC = 0.81 ± 0.02% - 0.83 ± 0.01%), while average OA and k hardly changed (OA = 79.9 ± 0.02% - 76.6.4 ± 0.03%, kappa = 0.48 ± 0.02–0.45 ± 0.02), indicating that model performance was not strongly dependent on sample size (Fig. 8a and b). Interestingly, the coupled PSNFT-spread model yielded more accurate disease distribution maps than the PSFNT model alone, irrespective of sampling sizes.

The PSNFT model and the coupled PSNFT-spread model, which yielded the best performances, were also assessed at the orchard level (supplementary Table S8). When the RS-based PSNFT model was built with the particular conditions of each orchard, the accuracy of the RS-based PSNFT model improved (average OA = 81%); in fact, it exceeded the performance of the PSNFT-spread model for all orchards (average OA = 78–81% and average AUC = 0.83–0.84 for the different sample size). The analyses at orchard level indicated that model accuracy largely depends on the relative proportion of asymptomatic vs *Xf*-symptomatic trees. In general, when the proportion of symptomatic trees was higher, the PSNFT model performed best (OA = 77–85% and AUC = 0.62–0.82). Conversely, when the proportion of asymptomatic trees was greater, the coupled PSNFT-spread model performed better (OA = 77–95% and AUC = 0.67–0.98). However, this trend was not observed in orchards 1 and 4, in which both models showed a lower performance, estimated as OA = 68–79%, kappa = 0.35–0.50 and AUC = 0.67–0.72, respectively, for the PSNFT model, and OA = 74–79%, kappa = 0.43–0.58 and AUC = 0.79–0.86, respectively, for the coupled PSNFT spread model.

**Fig. 9:**
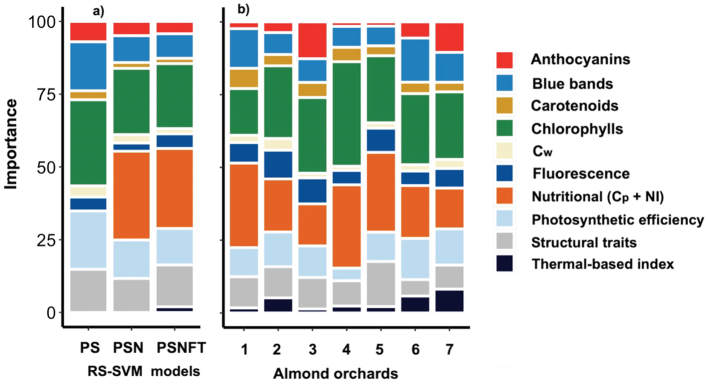
Plant-trait contribution for detecting *Xylella fastidiosa* symptoms for the RS-based SVM models (PS, PSN, PSNFT) using the training samples (a) and at orchard scale for the PSNFT model (b). In (a), each RS-based SVM model refers to i) pigment- and structure-based functional traits (PS), ii) pigment-structure and nutritional traits (PSN) and iii) pigment, structure, nutritional-fluorescence and thermal-based functional traits (PSNFT). The importance analysis was conducted using the average of training samples (*n* = 1091 almond trees).

We estimated predictor importance in the PS, PSN and PSNFT-SVM models to assess the usefulness of the plant traits to detect *Xf* symptoms (Fig. 9a; supplementary Table S9). However, the orchards differed in the composition of almond varieties, physiological status, water stress regimes and nutritional deficiencies, all of which may have influenced the detectability of *Xf* symptoms. Therefore, we also estimated the importance of each predictor at the orchard level (Fig. 9b; supplementary Table S10) and using the qPCR samples (*n* = 318 trees; supplementary Table S9) for the PSNFT-SVM model, which had the complete set of plant traits. The analysis conducted in the PS model revealed that the chlorophyll indicators from reflectance bands (i.e., TCARI, NPQI, VOG_2_ and D_att_-CabC_x_ + c) and the chlorophyll content retrieved from RTM inversion were the plant traits that contributed most to this model, reaching up to 30%. These were followed by indicators related to photosynthetic efficiency-xanthophyll cycle (PRI_n_, PRI_M1_), blue bands (BRI_2_), structural traits (LAI and LIDF_a_) and anthocyanins, with a contribution of 20%, 16%, 15% and 7%, respectively. When leaf protein content and nitrogen indices (RSI, CI_2_, GnyLi and MCARI_1510_) were included in the model, the weight of chlorophyll decreased to 23–22% for the PSN and PSNFT models respectively. The leaf protein content and NIs along with chlorophyll pigments contributed most in the PSN model (See supplementary Table S8). Furthermore, when leaf protein content and NIs were included in the model, the contribution of the structural traits and anthocyanin pigments decreased to 11% and 5%, respectively. When the thermal-based water stress trait (T_c_) and FLD_2_-based chlorophyll were included in the PSNFT model, the remaining plant traits reduced their contribution; with the exception of structural traits, which increased to 14%. The leaf protein content and nutritional indices contributed 27% (Fig. 9a), compared to 7% for SIF (5%) and T_c_ (2%) together. When we conducted the analysis at the orchard level (Fig. 9b), we found that the plant trait contribution varied between orchards. These variations between functional plant traits followed the same trend as when we globally analyzed the PSNFT model (Fig. 9a).

### 3.4 RS-based SVM model coupled with a stochastic epidemic spread model for early detection of *Xylella fastidiosa* in almond trees

We assessed the PSNFT model and the coupled PSNFT-spread model against the presence of *Xf* infection determined visually and also by qPCR test (*n* = 318 trees) on samples collected in the orchards. (Table 3). In most orchards, the coupled PSNFT-spread model classified the greatest number of trees correctly with respect to the qPCR tests. In general, the fraction of well-classified trees was higher for the coupled PSNFT-spread model (72% for a sample size of 5%) than for the visual (64%) or PSNFT model (63%). We further explored whether, compared to the PSFNT model, the RS-spread model improved the detection of early symptoms at a pre-visual stage by (i) evaluating the accuracy of its disease distribution estimations using qPCR assay data (Table 4); and (ii) counting the number of trees identified as *Xf*-symptomatic by the RS-spread model and confirmed by qPCR assay data but showing no visible *Xf* symptoms at field level.

**Table 3:** Number of almond trees with different disease status and tested by qPCR to determine *Xylella fastidiosa* infection and percentage of trees correctly classified by the PSNFT model and the coupled PSFNT-spread model at orchard levels (*n*_total_ = 1426 trees). The orchards located in the same area were grouped to simplify the analysis.

	1	2	3	4	5	6	7	8	Total
Asymptomatic: DS = 0	63	26	81	299	23	95	32	38	657
Symptomatic: DS ≥ 1	52	77	151	166	56	155	83	30	769
qPCR	39	35	31	72	19	91	28	3	318
Negative	9	4	10	16	1	31	1	1	73
Positive	30	31	21	56	18	60	27	2	245

% well-classified trees according to the qPCR assay									
Visual (*n* = 318)	62	66	84	55	63	58	86	100	65
PSNFT	56	68	81	52	64	57	90	33	62
PSNFT+ spread (5%)	71	79	99	59	68	65	91	53	72
PSNFT+ spread (10%)	65	78	99	59	68	59	83	47	68
PSNFT+ spread (15%)	66	64	99	57	68	56	73	33	64
PSNFT+ spread (20%)	68	62	97	56	68	57	72	33	64

**Table 4:** Assessment of the model predictions (OA and kappa) for early detection of *Xylella fastidiosa* symptoms (DS = 0–1) vs. qPCR assay (n = 318 trees). ‘Asymptomatic infections’ (AF) refers to the proportion of trees classified as symptomatic by the PSNFT model and the coupled PSNFT-spread model that showed no visual symptoms in the field (DS = 0; *n* = 168 trees) but were positive by qPCR (*n* = 105 trees).

RS-models/Visual inspection	OA	kappa	*AF*
Visual inspection	64.5%	0.31	–
PSNFT	63.4%	0.26	17%
PSNFT+ spread (5%)	71.7%	0.33	58.6%
PSNFT+ spread (10%)	68.3%	0.32	56.1%
PSNFT+ spread (15%)	64.4%	0.25	47.6%
PSNFT+ spread (20%)	64.4%	0.23	46.2%

The assessment of the PSNFT-spread model validated with the tree-level qPCR dataset yielded an average OA and kappa that ranged between 64 and 72% and 0.23–0.33, respectively, for the five sample sizes (depending on sample size, Table 4). The performance of the PSNFT (OA 63% and kappa 0.26) and the visual inspection (OA 64% and kappa 0.1) showed a lower accuracy compared to *Xf*-infection status determined by qPCR, indicating a lower efficacy than the combined PSNFT-spread model.

At orchard level, we focused on orchards 1 and 4, which showed the lowest disease incidence levels (~45% and ~ 36% of diseased trees, respectively). This was done because these orchards lowered the performance of the PSFNT models at orchard level; thus, it was thought that they could provide insights into the limited performance and that the implementation of early detection would be more relevant in these orchards. The performance of the PSNFT-spread model at orchard level (Table 3) verified with the tree-level qPCR dataset yielded an average OA ranging between 64% and 72% for the range of sample sizes, and 71% and 59% for orchards 1 and 4, respectively. However, when compared against the *Xf*-infection status confirmed by qPCR, the PSNFT model (OA of 56% and 52% for orchard 1 and 4) and the visual inspection (OA of 61% and 55% for orchard 1 and 4) had lower accuracies than the PSNFT-spread model.

**Fig. 10:**
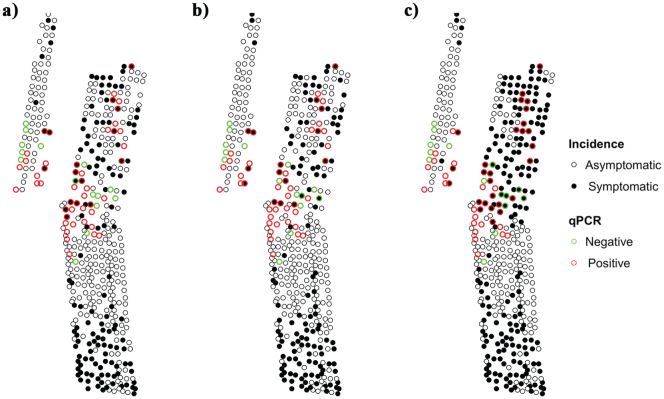
Location of almond trees in orchard 4 affected by leaf scorch caused by *Xylella fastidiosa* (*Xf*) according to their disease status (asymptomatic vs. symptomatic after visual inspection) and *Xf*-infection status determined by qPCR test (a), predicted by the RS-PSFNT model (b) and estimated by the PSNFT coupled with the stochastic spread model using a 5% sampling size (c).

The disease distribution maps generated from the PSNFT-spread model improved the detection of *Xf*-infected asymptomatic trees (Fig. 10c) that did not show visible symptoms (Fig. 10a) and were missed by the PSNFT model (Fig. 10b). The PSNFT-spread model detected asymptomatic *Xf*-infected trees (qPCR = 1; DS = 0) with an overall accuracy of 55–80% and 36–39% for the range of sample sizes and for orchards 1 and 4, respectively. By contrast, the PSNFT model yielded an OA of 17% and 3% for orchards 1 and 4.

## 4 Discussion

### 4.1 Leaf-level X*ylella fastidiosa* assessment using physiological measurements

Leaf-level assessments showed that *Xf* symptoms induced spectral changes in the VNIR region due to the degradation of chlorophyll and carotenoids (Fig. 2, Fig. 3). The changes in chlorophyll and anthocyanins as *Xf* infection increased confirmed the previous results of Zarco-Tejada et al. (2018) in olive trees. The increase of anthocyanins when *Xf*-symptoms worsened is due to the protective role that anthocyanins play against damage induced by plant pathogens or environmental stress. We further found a strong link between N and chlorophyll at leaf level, which is widely supported in the scientific literature (e.g., Serrano et al., 2002; Herrmann et al., 2010; Tremblay et al., 2011; Homolová et al., 2013). Our results also agree with those of Purcino et al. (2007) and Ribeiro et al. (2004), who reported that *Xf* bacterium has a detrimental effect on the nitrogen metabolism and photosynthesis in orange leaves infected by *Xf.* When we compared the pigments and nitrogen levels with the qPCR assays and visual inspections, we found that chlorophyll and nitrogen levels dropped in symptomatic leaves sampled from non-*Xf*-infected trees (Fig. 3b and c). This may indicate that chlorosis or nitrogen deficiency symptoms alone are not specific of *Xf* infection and can be induced by other biotic or abiotic stress factors. This suggests that for *Xf*-symptoms to be successfully detected, pigments and nutritional indicators should be complemented with additional plant traits in order to distinguish *Xf*-symptoms from other stress conditions, such as water stress.

Reduction in stomatal conductance was also observed when *Xf* symptoms increased (Fig. 4a). Colonization of xylem vessels by *Xf* interferes with transpiration and leads to a decrease in stomatal conductance to the point of stomatal closure. This reduces the rate of photosynthesis and transpiration, preventing evaporative cooling and consequently increasing leaf temperature (Berni et al., 2009). This is in agreement with previous studies, which have found a relationship between *Xf* infection and water stress in orange trees (Ribeiro et al., 2004), olive trees (Zarco-Tejada et al., 2018) and Virginia creepers (*Parthenocissus quinquefolia*) (McElrone and Forseth, 2004). We hypothesized that the reduction in fluorescence (Fig. 4b) may have been due to a combination of photosystem photo-inhibition and the early onset of leaf senescence. In agreement with McElrone and Forseth (2004), this hypothesis was supported by the reduction of nitrogen content and chlorophyll pigments when anthocyanin production increased. These pigment changes in the leaves affected by *X. fastidiosa* initiated a photoprotective process due to the water stress derived from the *Xf* infection and the senescence of the leaves. This study conducted at leaf level show that stomatal conductance, leaf temperature, pigments (C_ab_ and A_nth_), nitrogen and chlorophyll fluorescence emission are valuable indicators to discriminate between asymptomatic and *Xf*-symptomatic leaves.

### 4.2 Plant traits and spectral indices to detect tree crowns with *Xylella fastidiosa*

The plant traits retrieved from model inversions, along with the spectral indices and thermal-based index, enabled the detection of *Xf* symptoms in almond trees. In particular, based on VIF analysis and Wilks' lambda tests, we identify the most parsimonious models in each spectral domain (VNIR and SWIR).

Our results suggested that photosynthetic indices, leaf protein content, nitrogen indices, pigments and water stress indicators all can contribute to *Xf* detection in almond trees. At crown scales, we also showed that anthocyanins gradually increased as *Xf* symptoms worsened (Fig. 7b; supplementary Fig. S7c). We highlight that inverted leaf protein content also tracked the differences between trees with and without *Xf* symptoms. This finding is in agreement with Purcino et al. (2007), who showed that *Xf* infection significantly changed the nitrogen metabolism of leaves (e.g., glutamine synthetase) and the accumulation of nitrogen compounds into the xylem.

Concerning narrow-band indices used in the PS model, we found that blue/red spectral indices (i.e.: BRI_2_) differed between asymptomatic trees and those showing either initial or advanced leaf scorch symptoms caused by *X. fastidiosa*. These results confirm those of previous studies that identified blue/red spectral indices for early detection of red leaf blotch caused by the fungus *Polystigma amygdalinum* in almond trees (López-López et al., 2016), Verticillium wilt in olive trees (Calderón et al., 2013, Calderón et al., 2015) and *X. fastidiosa* in olive trees (Zarco-Tejada et al., 2018; Poblete et al., 2020). The chlorophyll degradation-based spectral trait (NPQI) is related to the degradation of chlorophyll via phaeophytinization (Barnes et al., 1992; Penuelas et al., 1995). The PRI indices are associated with light use efficiency (LUE) at leaf scale (Guo and Trotter, 2004). Overall, The PRI_n_ normalized by crown chlorophyll (Fig. 6c) and the crown thermal-based trait (Fig. 7a) are inversely related to stomatal conductance and water potential (Zarco-Tejada et al., 2013a; Gonzalez-Dugo et al., 2019). A reduction in stomatal regulation, transpiration and photosynthetic rate caused by infection and colonization of xylem vessels by *X. fastidiosa* led to a strong decrease in fluorescence emission at leaf and crown scales (Figs. 4b and 6d, respectively). Therefore, indicators of gas exchange dynamics contribute strongly to early detection of *Xf* infection in almond trees.

Although, the NIR/SWIR-based nitrogen indices (MCARI_1510_, CI_2_, RSI, and GnyLi) had a coarse spatial resolution (80 cm/pixel), they were also capable of detecting *Xf* symptoms not only in advanced stages but also in early ones, with higher nitrogen values in asymptomatic trees than in symptomatic ones. The NIs used in the PSN and PSNFT-SVM models to detect *Xf* symptoms were based on bands close to the red-edge region (the CI_2_ and RSI), which is highly correlated with chlorophyll pigments (Gitelson et al., 2002). Other NIs were based on spectral bands associated with the nitrogen absorption peak (MCARI at 1510 nm). The GnyLi index uses spectral bands (900 nm and 1050 nm) related to biomass (Peñuelas and Fillela, 1998) and spectral bands (955 nm and 1220 nm) sensitive to plant moisture (Clevers et al., 2010). Reflectance at 1020 and 1510 nm responds particularly strongly to nitrogen content due to the first overtone of the N—H band vibration, generating an absorption feature of N and proteins (Curran 1989), and provides a useful nitrogen content proxy (e.g., Serrano et al., 2002; Herrmann et al., 2010).

### 4.3 RS and epidemic models for *Xylella fastidiosa* detection

The PSNFT model (Fig. 8), which combined nutritional traits (C_p_ and NIs) with T_c_ and solar-induced chlorophyll emission, improved the average overall accuracy of the predictions by around ~2% (OA ≥ 75%, kappa = 0.50), compared to PS model (OA ≥ 73%, kappa = 0.46), We found that coupling the epidemic spread model and the RS-based model increased accuracy by around 5% (OA = 80%, kappa = 0.48) compared to the PSNFT model. In comparison to the PS model based on plant traits in the VNIR, the coupled PSNFT-spread model increased accuracy by more than 7% in OA. These results highlighted the moderate improvements yielded by coupling the PSNFT model with the stochastic spread model for the most accurate detection of *Xf* infection.

In addition, we evaluated the parsimony of the main RS models (PS, PSN and PSNFT) adding more spectral indices not selected by VIF analysis in each RS-based SVM model. In all the cases analyzed, the proposed RS models were the most parsimonious ones (not shown for the sake of brevity). This fact indicates that adding more variables to the RS models does not automatically yield the most parsimonious model. Consequently, the use of the VIF-Wilks' test reduced overfitting in the RS models. Additionally, we assessed different combinations of the PS model by adding SIF (VNIR sensor), thermal-based T_c_ index and leaf protein content (measured with the NIR-100 sensor) in the PS model (supplementary Table S7). In general, when we added these indicators, the performance of the PS model increased. Nonetheless, in any case the performance was not superior to the PSNFT model. We noted that when the three indicators (SIF + T_c_ + C_p_) were added together to the PS model, the model had an average OA close to 75%. It is also worth noting that the PSNFT model without thermal information performed more poorly than the PSNFT model (OA = 74%, kappa = 0.48). However, it yielded higher performance than those models based only on pigments and structural parameters (supplementary Fig. S9 and supplementary Table S7). Overall, the RS models predict *Xf* symptoms better in almond trees when they included fluorescence, leaf protein content and thermal information as predictors.

Our results show that high-resolution SWIR imagery can help to quantify parameters related to leaf protein content and structural parameters that can improve *Xf*-detection compared to VNIR hyperspectral imagery alone. However, using high-resolution SWIR cameras in operational RS applications remains challenging because the higher cost of these sensors. In this context, it is worth noting that complementing a VNIR sensor with a thermal sensor can generate results similar to those from a more complex or costly sensor combination.

### 4.4 Coupling RS models with an epidemic model

The analysis at orchard level showed that, when the relative proportion of symptomatic trees was higher than that of asymptomatic ones, the RS-based SVM PSNFT model and the coupled PSNFT-spread model performed best (supplementary Table S8). This indicates that both models captured early and advanced symptoms of *Xf* infection particularly well. However, when the asymptomatic/*Xf-*symptomatic proportion changed towards asymptomatic trees (i.e., orchards 1 and 4), the accuracy of the PSNFT model and the coupled PSNFT-spread model decreased (supplementary Table S8). This phenomenon could be explained by the difficulties detecting visual symptoms at the very early stage of symptom development and the unusual high proportion of asymptomatic infections. This was confirmed when the visual inspections were compared to qPCR in a specific orchard, such as orchard 4 (DS_0_ = 299; DS_≥1_ = 166), where 70% of trees (31/56 trees) showed the existence of asymptomatic infections. Similarly, in orchard 1, the PSNFT model and the coupled PSNFT-spread model exhibited poor accuracy (OA = 77% for PSNFT model and OA = 76–79% for the coupled PSNFT-spread model), likely due to the high proportion (67%; 12/18 trees) of trees with asymptomatic *Xf* infection.

### 4.5 Plant trait contributions in RS-SVM models for the detection of *Xylella fastidiosa*

The importance analysis suggested that the most reliable functional traits to distinguish between asymptomatic and *Xf*-symptomatic trees were plant traits related to nutritional-pigment changes. In particular, the chlorophyll indicators made the greatest contribution in the PS model (30%). This is consistent with earlier studies focused on the VNIR domain (Zarco-Tejada et al., 2018; Poblete et al., 2020). We further highlight that pigment-nutritional functional plant traits (i.e., anthocyanins, chlorophylls, NIs and leaf protein content) represented more than 55% of the contributions in the PSNFT model, compared to photosynthesis, water-stress and photosynthetic efficiency plant traits, which reached ≃ 20%.

The variable importance analysis conducted at orchard level (supplementary Table S10) showed that the plant trait contribution varied among orchards. These differences could be associated with water stress, nutritional condition, photosynthesis activity and canopy structures of the orchards (Fig. 6, Fig. 7). The contribution of anthocyanins increased in orchards (i.e., orchard 3 and 7) with a greater ratio of trees with advanced *Xf* symptoms (DS ≥ 1) compared to asymptomatic trees (DS =0). When we analyzed the contribution of the plant traits in the subset of trees with qPCR tests (*n* = 318 trees; where 78% positive and 22% negative), we found that nutritional plant traits have a greater contribution than chlorophylls (supplementary Table S9). These findings suggest that monitoring the nitrogen available for remobilization among plant parts could be the key to detect changes associated with *Xf*-symptoms. This is in agreement with Purcino et al. (2007) who suggested that *Xf* symptoms could modulate the absorption, assimilation, and distribution of nitrogen in the host plant. Interestingly, structural indices made a smaller contribution to the orchard-level model than to the model that used 75% of the studied trees across orchards (supplementary Table S9). This finding was consistent with those obtained by Calderón et al. (2015) who found that structural indices were not selected by stepwise linear discriminant analysis to distinguish between Verticillium wilt severity levels in olive trees by SVM-based classification at orchard level. The non-selection of structural indices may be due to the fact that crown structure varies less within orchards than between orchards.

### 4.6 Uncertainties of the inverted plant traits in remote sensing models

Uncertainties in the plant traits inverted through biophysical modelling with RTMs, may propagate into predictive models relying on those traits. Here, we reduced the uncertainties of the RTM inversion by constraining parameters in the LUT table to realistic ranges. This eliminates unexpected parameter combinations and thus alleviates the ill-posed problem. The validation of chlorophyll and anthocyanin content at crown level with the leaf Dualex readings (supplementary Fig. S2 and S3) showed that the ML inversion minimized the uncertainty associated with the spectral response of the sensors, as well as atmospheric and background differences. The significant relationships obtained between the structural parameters (LAI and LIDFa) and narrow-band indices (NDVI and SIF) suggests that structural plant traits were adequately retrieved by biophysical modelling. However, without reference field measurements, the uncertainties of the structural traits are ultimately unknown, and may reduce the accuracy of the RS models. We highlight that the relation found between SIF and LIDFa is due that LIDFa and together with LAI and chlorophyll content determines the large variability of the chlorophyll fluorescence emission (Verrelst et al., 2015). It furthermore suggests that the SIF emission retrieved at coarse spectral resolution (6 nm) could be a mixture between SIF and structural parameters.

The RF inversions used the entire spectral range (400–1700 nm) of our measurements to retrieve C_p_ and C_w_. The maps of the Person's correlation with NDSI (supplementary Fig. S5) showed that the bands most correlated with C_p_ were those beyond 1500 and 1700 nm, which is the optimal subdomain for estimating leaf proteins as shown in Féret et al. (2021). C_w_, in turn, correlated significantly with bands placed in the subdomain of 1000–1700 nm, which is the spectral region for water absorption. Nevertheless, for the further improve estimates of leaf protein content, high-resolution is required to separate all influencing constituents in the 1500–1700 nm spectral subdomain, especially when upscaling machine learning methods from the leaf to the top of tree crowns level. We remark that the proposed machine learning approach could be valid for plant traits that we were not able to be validated with field data. Nevertheless, it is advisable for future research to take additional field measurements to quantify the unknow uncertainties of the machine learning inversions.

### 4.7 Early detection of *Xylella fastidiosa* through qPCR assays

The qPCR test revealed that the coupled PSNFT-spread model yielded a higher percentage of correctly classified trees than the PSNFT model or the visual inspection conducted in most orchards (Table 3). Overall, the performance of the PSNFT-spread model improved when the random sample was smaller. However, the visual inspections of symptoms reflected infection status quite well overall, with a true-negative rate of 87% (i.e., qPCR-negative trees classified as asymptomatic) and a true-positive rate of 58% (i.e., qPCR-positive trees classified as symptomatic).

Early detection of new infections is critical to mitigating the impact of *Xf* on crops. The PSNFT model did not guarantee the detection of early symptoms that visual inspections cannot yet identify. The combination of the PSNFT model with an explicit stochastic epidemic spread model improved the detection of infected trees at early stages of *Xf* disease development in the almond orchards studied. Moreover, when the spread model was coupled with the PS model for the same size sample (5%), in an orchard with low disease incidence (supplementary Fig. S10), the performance was lower (OA = 71% and kappa =0.41) than when it was coupled with the PSNFT model (OA = 74% and kappa = 0.46). This suggests that the adequate selection of plant traits in the RS models improve the accuracy of the coupled RS-spread model to detect *Xf* infection. The disease distribution maps estimated by the PSNFT-spread model yielded higher accuracies than those of the PSFNT model on its own for all sample sizes. In contrast to the PSFNT model, the PSNFT-spread model classification showed less sensitivity when compared to visual inspection (77% by the PSNFT and 75–77% by the RS-spread model with increasing sample size). This lower sensitivity in the classification may have been due to (i) error and uncertainty inherent to the RS-spread model to detect infected trees; and (ii) trees that were infected by *Xf* but showed no symptoms at the time of visual inspection.

We further explored the asymptomatic *Xf*-infected trees confirmed as symptomatic by qPCR assays. We found that trees that showed no visual symptoms but were confirmed as symptomatic trees via qPCR (*n* = 104) were detected with an accuracy of 16% by the PSFNT model. Yet, the PSNFT-spread model improved the accuracy of the predictions from 46% to 59% with a decrease in sample size. Thus, the coupled RS-spread model is particularly useful in orchards at early stages of disease development in which infected plants may be in the incubation period (i.e., pre-symptomatic infections). This was demonstrated in the *Xf* scenario of the south of Italy, where subsp. *pauca* was infecting olive trees (Calderón and Parnell, 2019). The integration of RS information in spread modelling demonstrated the accurate mapping of spatial correlations, typical for a vector-borne transmitted disease, and the capture of the effect of host spatial structure and landscape connectivity on disease distribution. These results obtained by RS-spread modelling and validated with qPCR data suggested that coupling the PSNFT with an epidemiological spread model improved the ability of the RS model to detect trees in the incubation asymptomatic period or at an early stage of the disease that cannot be detected by visual evaluations.

Future research should focus on the combination of remote sensing with spatial epidemic models in surveillance and the parameterization and design of predictive models for emerging epidemics. These models traditionally rely on parameters estimated from extensive epidemiological data collected via ground surveys and visual inspection, which are costly and non-exhaustive. The incorporation of remote sensing data into spatial epidemic modelling could be a step change in terms of the ability to quickly capture epidemic characteristics over vast areas. Moreover, given a parameterized model, it is possible to infer the effectiveness of different surveillance and disease management scenarios (Parnell et al., 2017). It is for all these reasons that well parameterized epidemiological models and optimization techniques based on remote sensing can be used to optimize surveillance programs in order to maximize performance and minimize the costs involved.

## 5 Conclusions

This study demonstrates that coupling a remote sensing model based on pigment, structural plant traits, leaf protein content, NIR-SWIR-based nitrogen indicators, fluorescence and thermal information with a stochastic epidemic *Xf-*spread model improved the detection of *Xylella fastidiosa* (*Xf*) in almond orchards under severe rainfed conditions. In comparison to the best RS-based SVM model, the coupled RS-based SVM-spread model increased overall accuracies by more than 5%. The promising results obtained with the coupled RS-spread models for the early detection of *Xf* highlighted the suitability of this methodology for assessing the symptoms caused by *Xf* and other plant pests at large scales.

The integration of RS estimations in spatial spread modelling also demonstrated the accurate mapping of spatial correlations, typical for a vector-borne transmitted disease. They improved pre-visual detection of *Xf* infection due to the capture of the effect of host spatial structure and landscape connectivity on disease distribution. This study makes progress in the surveillance of emerging plant diseases and provides the basis for future use of well-parameterized epidemiological models based on remote sensing data in surveillance programs.

## Credit author statement

**C.C, R.C., B.B.L., S.P., J.A.N.-C. P.J.Z.-T., and P.S.A.B.:** Conceptualization, Methodology **C.C., R.C., H.D., and Y.C.:** Software, Validation, **C.C., B.B.L., M.R.,:** Data Curation **C.C, R.C.:** Investigation, Formal analysis **B.B.L., S.P., J.A.N.-C., P.J.Z.-T. and P.S.A.B.:** Supervision, Project administration, Funding acquisition, Resources **C.C., R.C., J.A.N.-C., P.J.Z.-T. and P.S.A.B.:** Writing - Original Draft **All authors:** Writing - review & editing

## Declaration of Competing Interest

The authors declare that they have no known competing financial interests or personal relationships that could have appeared to influence the work reported in this paper.

## Appendix A Supplementary data

Supplementary material Image 1
