# Supplementary material for: Detection of Xylella fastidiosa in almond orchards by synergic use of an epidemic spread model and remotely sensed plant traits
Source: Remote Sens Environ. 2021 Jul;260:112420. doi: 10.1016/j.rse.2021.112420 (PMC8169955; doi:10.1016/j.rse.2021.112420)
Supplement: Supplementary file 1 — Supplementary material [file mmc1.docx]

Supplementary Material for

**Detection of *Xylella fastidiosa* in almond orchards by synergic use of an epidemic spread model and remotely sensed plant traits**

C. Camino^*1^, R. Calderón^2^, S. Parnell ^2^, H. Dierkes^1^, Y. Chemin^1^, M. Román-Écija, M. Montes-Borrego^3^, B.B. Landa^3^, J.A. Navas-Cortes^3^, P.J. Zarco-Tejada ^4,3^, P.S.A. Beck^1^

1 European Commission (EC), Joint Research Centre (JRC), Ispra, Italy

2. School of Environment and Life Sciences, University of Salford, Manchester, United Kingdom.

3 Instituto de Agricultura Sostenible (IAS), Consejo Superior de Investigaciones Científicas (CSIC), Cordoba, Spain.

4. School of Agriculture and Food, Faculty of Veterinary and Agricultural Sciences (FVAS), and Department of Infrastructure Engineering, Faculty of Engineering and Information Technology (FEIT), University of Melbourne, Melbourne, Victoria, Australia

Content of this file

**Table S1** showing the set-up used for hyperspectral cameras during the airborne campaign.

**Table S2** showing the most relevant spectral indices used in this study.

**Table S3** showing the skill scores of predicted plant traits with PROSAIL-PRO model.

**Table S4** showing the statistical scores of Kruskal-Wallis test and Wilcoxon post-hoc test with Bonferroni correction in the analysis of Dualex reading conducted at leaf level.

**Table S5** showing the statistical scores of Kruskal-Wallis test and Wilcoxon post-hoc test with Bonferroni correction in the analysis of spectral indices carried out at canopy level.

**Table S6** showing the statistical scores of Kruskal-Wallis test and Wilcoxon post-hoc test with Bonferroni correction in the analysis of main inverted plant-traits and thermal index at canopy level.

**Table S7** showing the skill scores (OA, kappa and AUC) obtained by the studied RS-SVM models with all almond trees.

**Table S8 s**howing the skill scores (OA, kappa and AUC) obtained by the PSNFT model and PSNFT-spread model at orchard level.

**Table S9** showing the average plant-trait contribution (in %) for the three main RS-SVM models (PS; PSN and PSNFT) for detecting *Xylella fastidiosa* symptoms using the general and qPCR datasets.

**Table S10** showing the average plant-trait contribution (in %) for the PSNFT model used for detecting *Xylella fastidiosa* symptoms at orchard level.

**Fig. S1** showing the leaves sampled with no visual symptoms and showing leaf scorch caused by *Xylella fastidiosa.*

**Fig. S2** showing scatterplots of inverted chlorophyll content against chlorophyll content and nitrogen balance index measured with Dualex device.

**Fig. S3** showing scatterplot of inverted anthocyanin content against anthocyanins measured with Dualex device.

**Fig. S4** showing scatterplots between inverted LAI and LIDFa against NDVI and chlorophyll fluorescence emission, respectively.

**Fig. S5** showing contour maps of the Person’s correlation obtained by LAI, LIDFa, leaf protein content and leaf equivalent water thickness against ﻿the NDSI.

**Fig. S6** showing boxplots of inverted LAI, NDVI LIDFa as a function of the tree-based disease severity.

**Fig. S7** showing boxplots of chlorophyll, carotenoid and anthocyanin content retrieved by inversion RF approach as a function of the tree-based disease severity.

**Fig. S8** showing boxplost of leaf equivalent water thicknes and leaf protein content retrieved by inversion RF approach as a function of the tree-based disease severity.

**Fig. S9** showing the skill scores (OA, kappa and AUC) obtained for the studied RS-based SVM models (PS, PSN, PSNFT) and PSN + crown temperature (T_c_).

**Fig. S10** showing the visual inspection and the disease predictions for the remote sensing PS model and coupled PS-spread model in almond trees placed at orchard 4.

**Table S1.** Specification and set-up used for VNIR linear array micro-hyperspec imager and NIR/100 linear array micro- hyperspec imager during the airborne campaign.

| Specification/Configuration | VNIR micro- hyperspec | NIR/100 micro- hyperspec |
| --- | --- | --- |
| Spectral range | 400-850 nm | 950-1750 nm |
| Spectral bands | 260 | 165 |
| Aperture | Focal/2.5 | Focal/2.5 |
| Bit depth | 12-bit | 16-bit |
| Spatial resolution | 30 cm/pixel | 80 cm/pixel |
| FWHM Slit Image | 6.4 nm | 6.05 nm |
| spectral sampling interval | 1.85 nm/pixel | 6.0 nm/pixel |
| Entrance Slit Width | 25 µm | 25 µm |
| Angular field of view | 49.82° | 38.6° |
| Frame rate | 50 fps | 50 fps |
| Integration time | 18 ms | 40 m |

**Table S2**. Most relevant spectral indices used in this study to distinguish between asymptomatic and symptomatic almond trees showing leaf scorch caused by *Xylella fastidiosa* using the VNIR and SWIR spectral domain from high-resolution hyperspectral sensors.

| **Indices** | **Equation** | **Reference** |
| --- | --- | --- |
| **Chlorophyll a+b indices** | | |
| Transf. Chl. Absorp. Rfl. Index /Opt. Soil-Adjusted Veg. Index | *TCARI/OSAVI = [3((R_700_-R_670_)-0.2 (R_700_-R_550_)(R_700_/R_670_))]*  */[(1+0.16)(R_800_−R_670_)/(R_800_+R_670_+0.16)]* | Haboudane et al. (2002) |
| Normalized Phaeophyt. Index | *NPQI=(R_415_-R_435_)/(R_415_+R_435_)* | Barnes et al. (1992) |
| Vogelmann Index | *VOG2=(R_734_-R_747_)/(R_715_+R_726_)* | Vogelmann et al. (1993) |
| Reflectance band ratio index | *Datt-CabCx+c= R_672_/((R_550_*(3*R_708_)))* | Datt (1998) |
| ***R/G/B indices (Hyperspectral VNIR imagers)*** | |  |
| Blue index | *BF1 =R_400_/R_410_* | Zarco-Tejada et al. (2018) |
| Blue red index | *BRI2 =R_450_/R_690_* | (Zarco-Tejada et al., 2013) |
| **Xanthophyll indices and photosynthetic efficiency** | | |
| Normalized Photochemical Reflectance I. | PRI_n_=(R_570_-R_531_)/(R_570_+R_531_)/ RDVI (R_700_/R_670_)  Where RDVI=(R_800_-R_670_)/sqrt(R_800_+R_670_) | Zarco-Tejada et al. (2013)  Roujean and Breon (1995) |
| Photochemical Reflectance Index | PRI_M1_=(R_512_-R_531_)/(R_512_+R_531_) | Hernández-Clemente et al. (2011) |
| ***Chlorophyll fluorescence (****Hyperspectral VNIR imagers****)*** | | |
| Reflectance Curvature Index | *CUR= (R_675_ R_690)_/R^2^_683_* | (Zarco-Tejada et al., 2000) |
| ﻿SIF | *﻿FLD2=d-Rb; where*  *d_=_L_762_; R=(L_762_−L_750_)/(E_762_–E_750_) and b=E_762_* | *Moya et al. (2014) and*  *Plascyk and Gabriel (1975)* |
| **Nitrogen and NIR/SWIR-based indices** | | |
| Mod. Chl. Abs. Rfl. I. at 1510 | MCARI_1510_= [(R_700_- R_1510_)-0.2 (R_700_-R_550_)] (R_700_/R_1510_) | Herrmann et al. (2010) |
| Ratio Spectral Index | RSI=R_990_/R_720_ | Bao et al. (2013) |
| Combined index at 850 nm | CI2= (R_736_-R_735_)* (R_850_+R_720_) | This study |
| GnyLi | GnyLi= (R_900_*R_1050_) (R_955_*R_1220_)/(R_900_*R_1050_) + (R_955_*R_1220_) | (Gnyp et al. (2014) |

**Table S3.** skill scores (r^2^, RMSE and MAE) calculated input and predicted plant traits with the random forest approach using PROSAIL-PRO model. The spectral range used for each plant trait during the inversion is also shown.

|  | Spectral range | r^2^ | RMSE | MAE |
| --- | --- | --- | --- | --- |
| PROSPECT-PRO |  |  |  |  |
| C_ab_ | 400-800 nm | 0.97 | 3.18 µg/cm^2^ | 2.33 µg/cm^2^ |
| C_ar_ | 400-800 nm | 0.92 | 1.23 µg/cm^2^ | 0.85 µg/cm^2^ |
| A_nth_ | 400-800 nm | 0.93 | 0.58 µg/cm^2^ | 0.46 µg/cm^2^ |
| C_w_ | 400-1700 nm | 0.9 | 1.08 mg/cm^2^ | 0.73 mg/cm^2^ |
| C_p_ | 400-1700 nm | 0.8 | 0.62 mg/cm^2^ | 0.50 mg/cm^2^ |
| CBC | 400-1700 nm | 0.88 | 1.93 mg/cm^2^ | 1.42 mg/cm^2^ |
| SAILH |  |  |  |  |
| LAI | 400-1700 nm | 0.97 | 0.17 m^2^/m^2^ | 0.11 m^2^/m^2^ |
| LIDFa | 400-1700 nm | 0.91 | 5.31 degrees | 4.34 degrees |

**Table S4.** Chi-squared statistic (χ^2^) and *P*-value obtained from Kruskal-Wallis test followed by the Wilcoxon post-hoc test with Bonferroni correction in the analysis of Dualex reading conducted at leaf samples (n_total_=2,534 leaf samples). Dualex readings classified into four categories based on *Xylella fastidiosa* (*Xf*) disease severity (DS) according to the visual inspections and the qPCR test. *Healthy* refers to leaf-level measurements carried out on asymptomatic leaves where the absence of *Xf* bacteria was confirmed (qPCR = negative) and visual inspections assigned DS=0; *Asympt*. refers to leaf-level measurements conducted on asymptomatic leaves in trees where the presence of *Xf* bacteria was confirmed by qPCR (positive) and DS ≥1; *Sympt.* refers to leaf-level measurements conducted on symptomatic leaves in trees where the presence of *Xf* bacteria was confirmed by both methods (qPCR = positive and DS ≥1); *Non-Xf-inf.* refers to leaf-level measurements conducted on asympt./sympt. leaves in trees where the visual inspection assigned DS ≥1, yet qPCR was negative.

|  | Anth. | Cab | NBI | Flav |
| --- | --- | --- | --- | --- |
| Kruskal-Wallis test |  |  |  |  |
| χ^2^ | 136.99 | 124.06 | 127.31 | 52.021 |
| *P*-value | < 2.2 e-16 | < 2.2 e-16 | < 2.2 e-16 | 2.96 e-11 |
| Wilcox Test (*P*-value) |  |  |  |  |
| Healthy -Non-*Xf*-infected | 0.338 | 0.0104 | 0.037 | 9.49 e-04 |
| Healthy -Asympt. *Xf*-infected | 4.17 e-04 | 0.07 | 0.522 | 5.55e-06 |
| Healthy – Sympt. *Xf*-infected | 8.93 e-06 | 1.85 e-10 | 6.43 e-08 | 0.497 |
| Non-*Xf*-inf. - Asympt. *Xf*-infected | 0.988 | 0.0027 | 8.29 e-04 | 7.02 e-06 |
| Non-*Xf*-inf. – Sympt. *Xf*-infected | 0.015 | 0.861 | 1.00 | 9.49 e-04 |
| Asympt. *Xf*-inf. - Sympt. *Xf*-infected | 1.48 e-30 | 1.41 e-25 | 6.58 e-27 | 1.55 e-06 |

**Table S5.** Chi-squared statistic (χ^2^) and *P*-value obtained from Kruskal-Wallis test followed by the Wilcoxon post-hoc test with Bonferroni correction in the analysis of spectral indices studied to evaluate the significant differences between asymptomatic tree crowns (DS = 0), tree crowns with *Xylella fastidiosa* (*Xf*) symptoms (1 ≤ DS ≤ 2.5), and tree crowns with advanced *Xf* symptoms (DS ≥ 2.5).

|  | 1 | 2 | 3 | 4 | 5 | 6 | 7 |
| --- | --- | --- | --- | --- | --- | --- | --- |
| BRI_2_ |  |  |  |  |  |  |  |
| Kruskal-Wallis test | 57.61 | 41.73 | 46.10 | 124.56 | 40.73 | 39.59 | 58.22 |
| χ^2^ | 4.59 e-11 | 8.67 e-10 | 9.75 e-11 | < 2.2e-16 | 1.43 e-09 | 2.53 e-09 | 2.27 e-13 |
| *P*-value |  |  |  |  |  |  |  |
| Wilcox Test (*P*-value) |  |  |  |  |  |  |  |
| DS_0-_ DS*_Xf_* _sympt_. | 0.042 | 0.307 | 0.245 | 1.86 e-10 | 0.071 | 0.056 | 1.74 e-04 |
| DS_0_- DS_adv._ *_Xf_* _sympt._ | 9.85 e-08 | 3.39 e-08 | 3.96 e-09 | 2.48 e-25 | 9.14 e-07 | 3.89 e-07 | 4.16 e-06 |
| DS*_Xf_* _sympt_.-DS_adv._ *_Xf_* _sympt._ | 9.59 e-12 | 4.08 e-10 | 8.24 e-11 | 1.54 e-12 | 1.70 e-10 | 1.15 e-09 | 9.56 e-15 |
| PRI_n_ |  |  |  |  |  |  |  |
| Kruskal-Wallis test |  |  |  |  |  |  |  |
| χ^2^ | 75.47 | 64.64 | 41.71 | 108.13 | 63.52 | 107.19 | 88.37 |
| *P*-value | < 2.2e-16 | 9.20e-15 | 8.77e-10 | < 2.2e-16 | 1.61 e-14 | < 2.2e-16 | < 2.2e-16 |
| Wilcox Test (*P*-value) |  |  |  |  |  |  |  |
| DS_0-_ DS*_Xf_* _sympt_. | 8.25e-05 | 2.33e-05 | 9.58 e-03 | 9.80 e-03 | 3.01 e-04 | 9.25 e-05 | 1.06 e-09 |
| DS_0_- DS_adv._ *_Xf_* _sympt._ | 7.03e-10 | 1.13e-07 | 1.01 e-06 | 1.69 e-22 | 1.43 e-08 | 1.00 e-16 | 4.15 e-07 |
| DS*_Xf_* _sympt_.-DS_adv._ *_Xf_* _sympt._ | 9.97e-17 | 3.11e-14 | 8.74 e-10 | 8.34 e-19 | 2.09 e-14 | 5.39 e-23 | 1.04 e-17 |
| SIF_2_ |  |  |  |  |  |  |  |
| Kruskal-Wallis test |  |  |  |  |  |  |  |
| χ^2^ | 14.96 | 8.28 | 9.95 | 8.22 | 8.75 | 68.68 | 7.03 |
| *P*-value | 0.0006 | 0.0159 | 0.0069 | 0.0164 | 0.0126 | 1.22e-15 | 0.0297 |
| Wilcox Test (*P*-value) |  |  |  |  |  |  |  |
| DS_0-_ DS*_Xf_* _sympt_. | 0.1073 | 0.5187 | 0.0340 | 0.3416 | 0.8345 | 0.041 | 0.2264 |
| DS_0_- DS_adv._ *_Xf_* _sympt._ | 0.00039 | 0.0140 | 0.1253 | 0.0564 | 0.0115 | 0.277 e-16 | 0.0625 |
| DS*_Xf_* _sympt_.-DS_adv._ *_Xf_* _sympt._ | 0.00960 | 0.0246 | 0.0164 | 0.0136 | 0.0115 | 1.476 e-11 | 0.0381 |
| TCARI |  |  |  |  |  |  |  |
| Kruskal-Wallis test |  |  |  |  |  |  |  |
| χ^2^ | 108.75 | 96.306 | 74.76 | 47.865 | 92.34 | 177.18 | 81.675 |
| *P*-value | 2.44 e-24 | 1.22 e-21 | 5.82 e-17 | 4.04e-11 | 8.86 e-21 | 3.36 e-39 |  |
| Wilcox Test (*P*-value) |  |  |  |  |  |  |  |
| DS_0-_ DS*_Xf_* _sympt_. | 1.43 e-05 | 6.57 e-05 | 1.83 e-04 | 2.38 e-03 | 2.89 e-04 | 7.76 e-10 | 0.127 |
| DS_0_- DS_adv._ *_Xf_* _sympt._ | 4.18 e-23 | 1.38 e-20 | 2.12 e-16 | 1.38 e-06 | 5.22 e-20 | 3.65 e-34 | 1.76 e-17 |
| DS*_Xf_* _sympt_.-DS_adv._ *_Xf_* _sympt._ | 8.18 e-15 | 2.65 e-13 | 9.14 e-10 | 1.63 e-10 | 2.04 e-13 | 1.14 e-24 | 7.54 e-16 |
| MCARI_1510_ |  |  |  |  |  |  |  |
| Kruskal-Wallis test |  |  |  |  |  |  |  |
| χ^2^ | 29.30 | 19.08 | 18.93 | 81.88 | 16.12 | 16.33 | 23.05 |
| *P*-value | 4.33e-11 | 7.19 e-05 | 7.73 e-05 | < 2.2e-16 | 0.00032 | 0.0002 | 9.853 e-06 |
| Wilcox Test (*P*-value) |  |  |  |  |  |  |  |
| DS_0-_ DS*_Xf_* _sympt_. | 0.0188 | 0.3138 | 0.4899 | 8.69 e-05 | 0.415 | 0.017 | 0.2861 |
| DS_0_- DS_adv._ *_Xf_* _sympt._ | 0.0009 | 0.0010 | 0.0005 | 4.32 e-18 | 0.002 | 0.063 | 0.0002 |
| DS*_Xf_* _sympt_.-DS_adv._ *_Xf_* _sympt._ | 4.10 e-08 | 1.16 e-05 | 1.89 e-05 | 5.75 e-11 | 0.18 e-05 | 0.0004 | 1.62 e-06 |

**Table S6.** Chi-squared statistic (χ^2^) and *P*-value obtained from Kruskal-Wallis test followed by the Wilcoxon post-hoc test with Bonferroni correction in the analysis of spectral indices studied to evaluate the significant differences between asymptomatic tree crowns (DS = 0), tree crowns with *Xylella fastidiosa* (*Xf*) symptoms (1 ≤ DS ≤ 2.5), and tree crowns with advanced *Xf* symptoms (DS ≥ 2.5). Asterisk denotes that the statistical significance of the mean groups were evaluated according to the Tukey’s test due the normality of the residuals.

|  | 1 | 2 | 3 | 4 | 5 | 6 | 7 |
| --- | --- | --- | --- | --- | --- | --- | --- |
| Cab |  |  |  |  |  |  |  |
| Kruskal-Wallis test |  |  |  |  |  |  |  |
| χ^2^ | 23.24 | 23.33 | 26.13 | 81.41 | 19.77 | 31.44 | 21.17 |
| *P*-value | 8.99 e-06 | 8.55 e-06 | 2.11 e-06 | 2.10 e-18 | 5.09 e-05 | 1.49 e-07 | 2.53 e-05 |
| Wilcox Test (*P*-value) |  |  |  |  |  |  |  |
| DS_0-_ DS*_Xf_* _sympt_. | 7.48 e-06 | 7.88 e-06 | 3.44 e-06 | 4.14 e-16 | 7.34 e-05 | 1.44 e-07 | 2.54 e-05 |
| DS_0_- DS_adv._ *_Xf_* _sympt._ | 0.018 | 0.02 | 0.33 | 3.57 e-11 | 0.010 | 4.63 e-03 | 0.018 |
| DS*_Xf_* _sympt_.-DS_adv._ *_Xf_* _sympt._ | 0.338 | 0.322 | 2.30 e-03 | 0.135 | 0.866 | 0.825 | 0.576 |
| Anth |  |  |  |  |  |  |  |
| Kruskal-Wallis test |  |  |  |  |  |  |  |
| χ^2^ | 121.03 | 153.17 | 56.99 | 101.19 | 108.27 | 132.42 | 89.29 |
| *P*-value | 5.23 e-27 | 5.48 e-34 | 4.20 e-13 | 1.06 e-22 | 3.08 e-24 | 1.76 e-29 | 084e-20 |
| Wilcox Test (*P*-value) |  |  |  |  |  |  |  |
| DS_0-_ DS*_Xf_* _sympt_. | 9.22 e-12 | 7.11 e-17 | 1.06 e-01 | 8.17 e-11 | 5.94 e-10 | 3.004 e-12 | 2.76 e-05 |
| DS_0_- DS_adv._ *_Xf_* _sympt._ | 1.00 e-25 | 9.364 e-30 | 4.98 e-14 | 4.45 e-23 | 8.27 e-24 | 1.17 e-27 | 2.77 e-21 |
| DS*_Xf_* _sympt_.-DS_adv._ *_Xf_* _sympt._ | 3.13 e-07 | 8.39 e-07 | 3.55 e-09 | 9.79 e-06 | 1.52 e-06 | 9.82 e-09 | 4.63 e-09 |
| Cp |  |  |  |  |  |  |  |
| Kruskal-Wallis test |  |  |  |  |  |  |  |
| χ^2^ | 7.57 | 12.53 | 4.75 | 13.59 | 4.96 | 21.09 | 7.38 |
| *P*-value | 0.022 | 0.0019 | 0.092 | 0.00112 | 0.084 | 2.62 e-05 | 0.0249 |
| Wilcox Test (*P*-value) |  |  |  |  |  |  |  |
| DS_0-_ DS*_Xf_* _sympt_. | 0.969 | 0.006 | 0.579* | 0.022 | 0.99* | 0.46 | 0.0402 |
| DS_0_- DS_adv._ *_Xf_* _sympt._ | 0.0165 | 0.245 | 0.003* | 0.001 | 0.072* | 6.46 e-05 | 0.678 |
| DS*_Xf_* _sympt_.-DS_adv._ *_Xf_* _sympt._ | 0.0166 | 0.006 | 0.032* | 0.067 | 0.079* | 1.97 e-05 | 0.080 |
| LAI |  |  |  |  |  |  |  |
| Kruskal-Wallis test | 113.31 | 104.52 | 63.56 | 2.97 | 73.18 | 157.081 | 64.32 |
| χ^2^ |  |  |  |  |  |  |  |
| *P*-value | 2.49 e-25 | 2.01e-23 | 1.58 e-14 | 0.226 | 1.28 e-16 | 7.76 e-35 | 1.07 e-14 |
| Wilcox Test (*P*-value) |  |  |  |  |  |  |  |
| DS_0-_ DS*_Xf_* _sympt_. | 1.55 e-23 | 7.32 e-22 | 1.67 e-12 | 0.104 | 3.11 e-15 | 3.34 e-31 | 2.14 e-13 |
| DS_0_- DS_adv._ *_Xf_* _sympt._ | 7.49 e-11 | 1.59 e-09 | 6.46 e-08 | 0.496 | 9.75 e-08 | 8.29 e-15 | 5.17 e-07 |
| DS*_Xf_* _sympt_.-DS_adv._ *_Xf_* _sympt._ | 0.569 | 0.444 | 0.508 | 0.902 | 0.871 | 0.848 | 0.998 |
| Thermal |  |  |  |  |  |  |  |
| Kruskal-Wallis test |  |  |  |  |  |  |  |
| χ^2^ | 78.09 | 58.01 | 65.77 | 72.962 | 57.88 | 91.878 | 66.88 |
| *P*-value | 1.10 e-17 | 2.53 e-13 | 5.21 e-15 | 1.43 e-16 | 2.69 e-13 | 1.12 e-20 | 2.99 e-15 |
| Wilcox Test (*P*-value) |  |  |  |  |  |  |  |
| DS_0-_ DS*_Xf_* _sympt_. | 7.26 e-08 | 2.51 e-06 | 5.15 e-08 | 3.98 e-01 | 9.52 e-06 | 2.25 e-05 | 1.22 e-08 |
| DS_0_- DS_adv._ *_Xf_* _sympt._ | 2.94 e-07 | 1.06 e-05 | 3.53 e-05 | 2.05 e-15 | 4.66 e-06 | 1.12 e-12 | 5.01 e-05 |
| DS*_Xf_* _sympt_.-DS_adv._ *_Xf_* _sympt._ | 7.52 e-16 | 4.39 e-12 | 2.89 e-13 | 2.77 e-14 | 1.54 e-12 | 4.03 e-20 | 8.52 e-13 |

**Table S7**. Average skill scores (OA, kappa and AUC) obtained by the RS-SVM models for early detection of *Xylella fastidiosa* symptoms (DS = 0-1).

| **RS-models** | **OA** | **Kappa** | ***AUC*** |
| --- | --- | --- | --- |
| PS (pigments, C_w_, LAI, LIDFa, VNIR-indices) | 73.42 % | 0.46 | 0.73 |
| PSF (PS + SIF) | 74.34 % | 0.48 | 0.74 |
| PST (PS + T_c_) | 74.06 % | 0.48 | 0.74 |
| PSFT (PS + SIF and T_c_) | 74.69 % | 0.49 | 0.75 |
| PSN (PS + only C_p_) | 74.35 % | 0.48 | 0.74 |
| PSNFT (PS + only C_p_ + SIF and T_c_) | 74.88 % | 0.49 | 0.75 |
| PSNFT (PS + C_p_ + Nitrogen indices + SIF and T_c_) | 75.20 % | 0.50 | 0.75 |
| PSNT (PS + C_p_ + Nitrogen indices + T_c_) | 74.32 % | 0.48 | 0.74 |
| PSNFT+ spread (5%) | 79.85 % | 0.48 | 0.81 |
| PSNFT+ spread (10%) | 78.35 % | 0.46 | 0.82 |
| PSNFT+ spread (15%) | 77.28 % | 0.46 | 0.82 |
| PSNFT+ spread (20%) | 76.64 % | 0.45 | 0.83 |

**Table S8.** Overall accuracy (OA; %) kappa and area under the curve (AUC) obtained when classifying asymptomatic (DS=0) vs *Xylella fastidiosa* (*Xf*)-symptoms trees (DS ≥1 using the PSNFT model at orchard levels (n total= 1359 trees) and coupled PSNFT-spread model. The SVM algorithm was performed using 75% of trees of individual orchards, the remaining 25% was used for the evaluation. The coupled PSNFT-spread model was tested using multiple random sample placements at a range of sample sizes from 5 to 20% (step = 5). The orchards placed in same perimetral area were grouped for the analysis. The total number of asymptomatic trees and *Xf*-symptoms trees (DS ≥1) is also shown. Almond orchards with ≤ 75 trees were discarded from the analysis.

|  | **1** | **2** | **3** | **4** | **5** | **6** | **7** | **Avg.** |
| --- | --- | --- | --- | --- | --- | --- | --- | --- |
| n trees (DS_0_) | 63 | 26 | 81 | 299 | 23 | 95 | 32 | … |
| n trees (DS_1_) | 52 | 77 | 151 | 166 | 56 | 155 | 83 | … |
| OA |  |  |  |  |  |  |  |  |
| PSNFT | 68.26 % | 76.82 % | 83.69 % | 78.51 % | 84.51% | 79.08 % | 83.88 % | 79.49 % |
| PSNFT + SP_5%_ | 79.45 % | 76.12 % | 92.73 % | 73.62 % | 80.80 % | 82.01 % | 89.72 % | 80.89 % |
| PSNFT + SP_10%_ | 78.25 % | 77.42 % | 93.20 % | 72.65 % | 82.82 % | 74.91 % | 90.29 % | 79.49 % |
| PSNFT+ SP_15%_ | 75.10 % | 74.55 % | 92.89 % | 71.62 % | 81.79 % | 72.68 % | 91.63 % | 78.25 % |
| PSNFT+ SP_20%_ | 76.08 % | 73.40 % | 94.30 % | 70.98 % | 80.63 % | 71.30 % | 91.95 % | 77.97 % |
| kappa |  |  |  |  |  |  |  |  |
| PSNFT | 0.35 | 0.57 | 0.63 | 0.50 | 0.61 | 0.55 | 0.58 | 0.50 |
| PSNFT + SP_5%_ | 0.58 | 0.22 | 0.84 | 0.46 | 0.45 | 0.43 | 0.69 | 0.53 |
| PSNFT + SP_10%_ | 0.56 | 0.22 | 0.85 | 0.45 | 0.51 | 0.32 | 0.72 | 0.51 |
| PSNFT+ SP_15%_ | 0.50 | 0.21 | 0.84 | 0.44 | 0.48 | 0.32 | 0.78 | 0.50 |
| PSNFT+ SP_20%_ | 0.52 | 0.22 | 0.87 | 0.43 | 0.44 | 0.27 | 0.78 | 0.50 |
| AUC |  |  |  |  |  |  |  |  |
| PSNFT | 0.67 | 0.62 | 0.82 | 0.72 | 0.80 | 0.77 | 0.77 | 0.74 |
| PSNFT + SP_5%_ | 0.86 | 0.75 | 0.98 | 0.79 | 0.78 | 0.78 | 0.95 | 0.84 |
| PSNFT + SP_10%_ | 0.83 | 0.72 | 0.98 | 0.81 | 0.79 | 0.79 | 0.95 | 0.84 |
| PSNFT+ SP_15%_ | 0.81 | 0.66 | 0.98 | 0.81 | 0.76 | 0.79 | 0.96 | 0.83 |
| PSNFT+ SP_20%_ | 0.81 | 0.72 | 0.98 | 0.82 | 0.75 | 0.78 | 0.96 | 0.84 |

**Table S9.** Average plant-trait contribution (in %) for the three main RS-SVM models (PS; PSN and PSNFT) for detecting *Xylella fastidiosa* symptoms. The importance analysis was conducted using the average of training samples (n = 1,091 almond trees). The contribution of the plant traits using the qPCR samples (n= 318 almond trees) is also shown.

| **Plant traits** | **PS** | **PSN** | ***PSNFT*** | ***PSNFT _qPCR samples_*** |
| --- | --- | --- | --- | --- |
| Anthocyanins | 6.99 % | 4.91 % | 4.23 % | 2.62 % |
| Blue-bands | 16.83 % | 9.24 % | 8.46 % | 8.10 % |
| Carotenoids | 3.09 % | 1.98 % | 1.78 % | 2.44 % |
| Chlorophylls | 29.60 % | 22.79 % | 22.34 % | 14.59 % |
| Cw | 3.84 % | 2.88 % | 1.81 % | 2.12 % |
| Fluorescence | 4.75 % | 2.79 % | 4.98 % | 9.44 % |
| Nutritional (C_p_ +NI) | … | 30.50 % | 27.50 % | 29.49 % |
| Photosynthetic efficiency | 20.05 % | 13.22 % | 12.57 % | 16.90 % |
| Structural traits | 14.84 % | 11.69 % | 14.37 % | 10.49 % |
| Thermal-based index | … | … | 1.94 % | 3.90 % |

**Table S10.** Average plant-trait contribution (in %) for the PSNFT model used for detecting *Xylella fastidiosa* symptoms at individual orchards. The importance analysis was conducted using the average of training samples of each studied orchards.

| **Plant traits** | **1** | **2** | ***3*** | ***4*** | ***5*** | ***6*** | ***7*** |
| --- | --- | --- | --- | --- | --- | --- | --- |
| Anthocyanins | 2.3 % | 3.7 % | 12.7 % | 1.6 % | 1.5 % | 5.6 % | 10.5 % |
| Blue-bands | 13.6 % | 7.5 % | 8.2 % | 7.2 % | 6.8 % | 15.2 % | 10.3 % |
| Carotenoids | 6.9 % | 3.9 % | 5.1 % | 4.9 % | 3.4 % | 3.9 % | 3.3 % |
| Chlorophylls | 16.1 % | 25.0 % | 26.0 % | 35.9 | 23.1 % | 24.5 % | 23.2 % |
| Cw | 2.5 % | 4.0 % | 1.6 % | 1.4 % | 1.8 % | 2.2 % | 3.1 % |
| Fluorescence | 7.1 % | 9.9 % | 8.9 % | 5.0 % | 8.4 % | 5.0 % | 6.7 % |
| Nutritional (C_p_ +NI) | 29.0 % | 18.2 % | 14.5 % | 28.6 % | 27.4 % | 18.2 % | 14.0 % |
| Photosynthetic efficiency | 10.0 % | 11.9 % | 10.9 % | 4.3 % | 10.1 % | 14.1 % | 12.5 % |
| Structural traits | 10.7 % | 10.7 % | 10.8 % | 8.7 % | 15.5 % | 5.7 % | 8.3 % |
| Thermal-based index | 1.7 % | 5.2 % | 1.3 % | 2.3 % | 2.1 % | 5.8 % | 8.2 % |


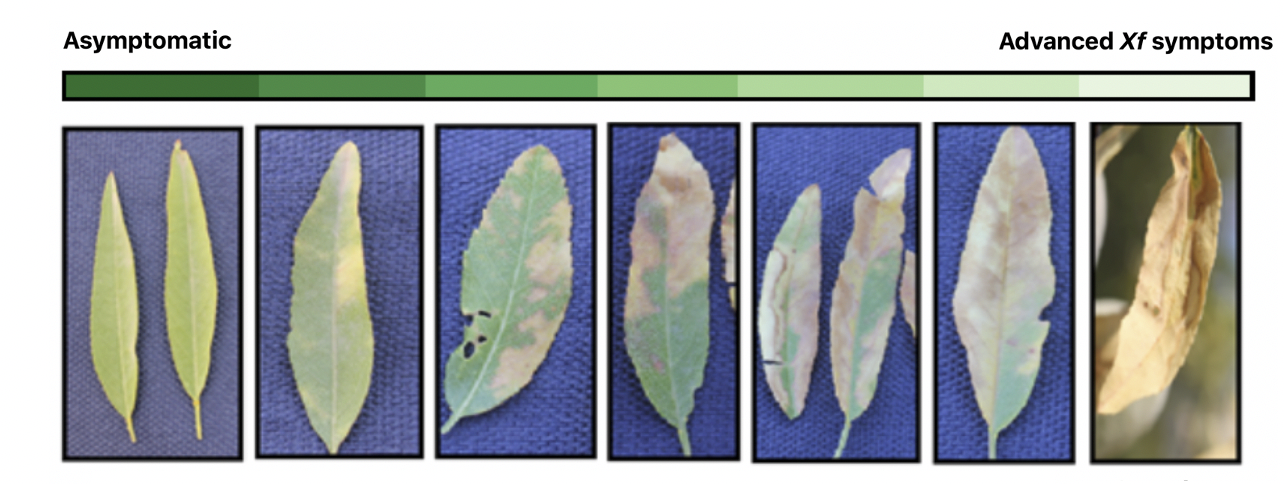


**Fig. 1.** Leaves sampled with no visual symptoms and showing leaf scorch caused by *Xylella fastidiosa* (*Xf*), from the first symptoms to a leaf completely affected by *Xf* symptoms.


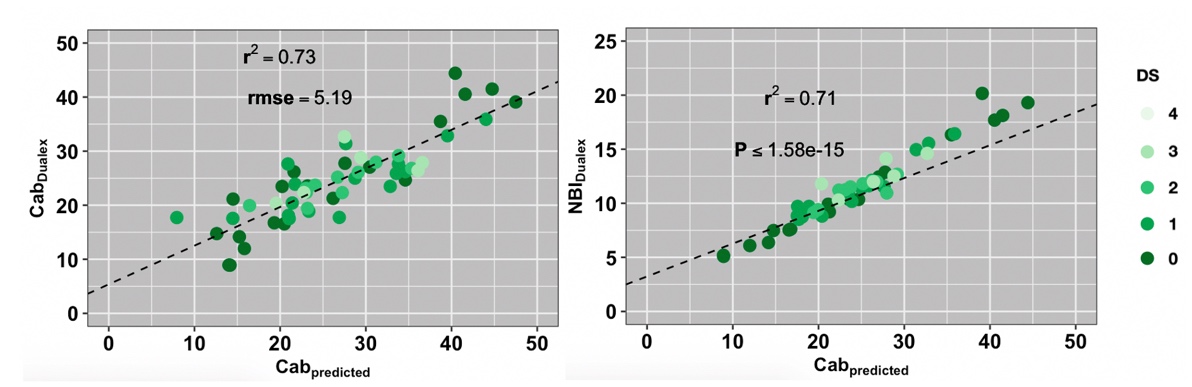


**Fig. S2**. Scatterplots of inverted chlorophyll content (in µg/cm^2^) against chlorophyll content (in µg/cm^2^) (a) and nitrogen balance index (NBI) measured (b) with Dualex device grouped by severity level caused by *Xylella fastidiosa*. The linear regression for dicots developed by Cerovic et al. (2012) was applied to recalibrate the chlorophyll estimations with Dualex device.


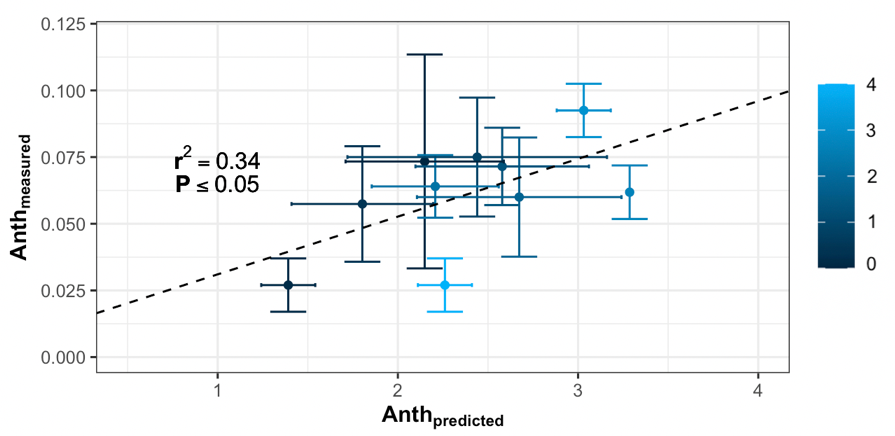


**Fig. S3**. Scatterplot of average anthocyanin content (in µg/cm^2^) inverted using PROSAIL-PRO model with anthocyanins (arbitrary units) measured with the Dualex device. Each point is the average aggregated by disease severity level caused by *Xylella fastidiosa*. The error bars denote the standard deviation of the data.


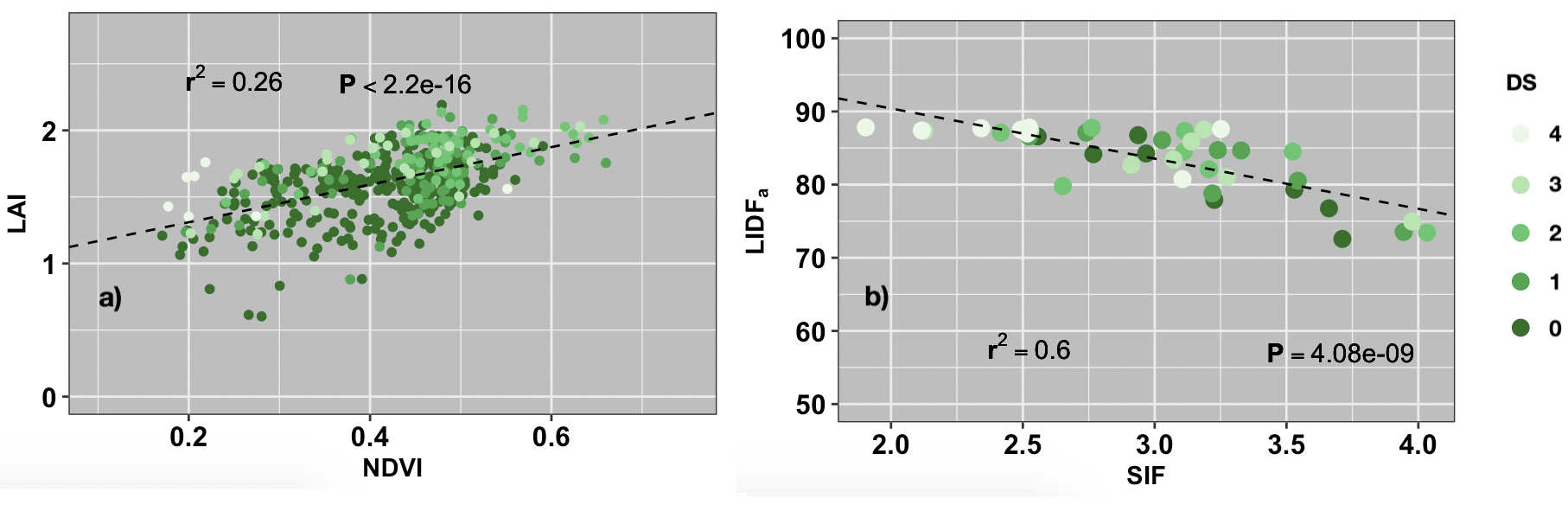


**Fig. S4**. Scatterplot of inverted LAI (in m^2^/m^2^) vs NDVI (a) as a function of the tree-based disease severity (DS) rating scale (0-4) in a random selected orchard. The scatterplot (b) between inverted LIDFa and chlorophyll fluorescence emission (SIF) from tree crowns grouped by plot and coloured by *Xylella fastidiosa* disease severity level.


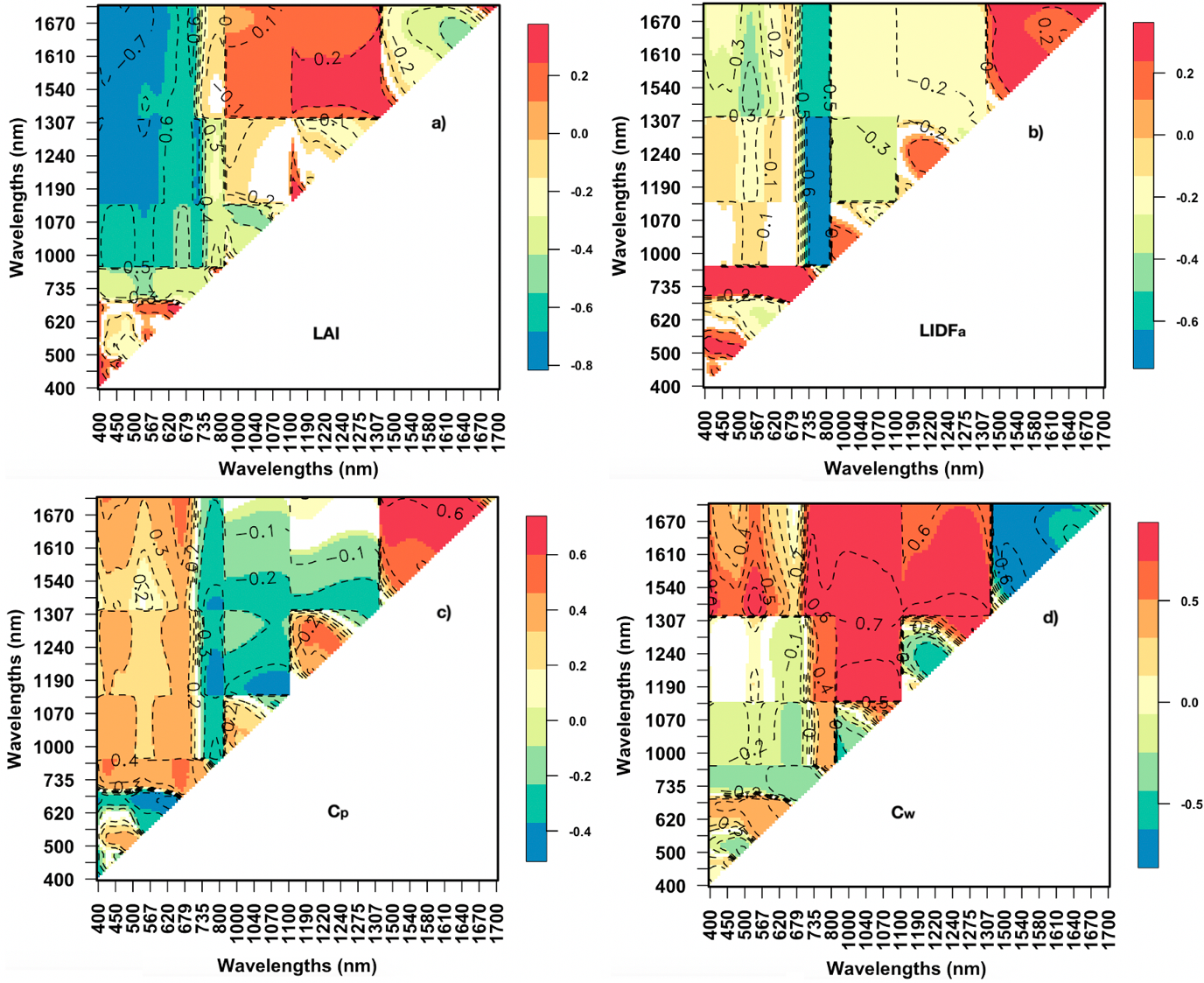


﻿ **Fig. S5**. Contour maps of the Person’s correlation obtained by LAI (a; in m^2^/m^2^), LIDFa (b; in degrees), leaf protein content (c; C_p_ in mg/cm^2^) and leaf equivalent water thickness (d; C_w_ in mg/cm^2^) retrieved using biophysical modelling against ﻿the normalized difference spectral index (NDSI) using all hyperspectral reflectance combinations of two wavebands at i and j nm. ﻿The NDSI is defined as: NDSI=(ρ_i_-ρ_j_)/ =(ρ_i_+ρ_j_); where ρ is hyperspectral reflectance at i and j nm.


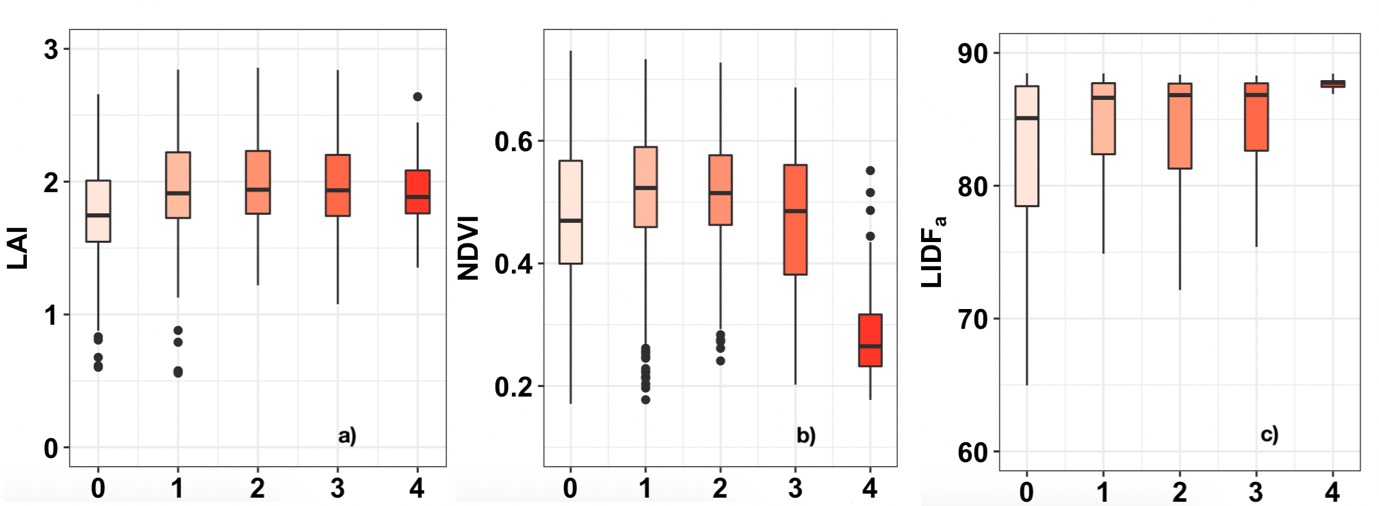


**Fig. S6**. Boxplots of inverted LAI (a; in m^2^/m^2^), NDVI (b) inverted LIDFa (a, in degrees) as a function of the tree-based disease severity (DS) rating scale (0-4). Leaf angle degrees of 0 correspond to planophile configurations and 90 degrees correspond to erectophile configurations. For the analysis, we used 1,426 almond trees grouped by DS class: where DS_0_ = 657 trees, DS_1_ = 359 trees, DS_2_ = 214 trees, DS_3_ = 142 trees and DS_4_ = 54 trees).


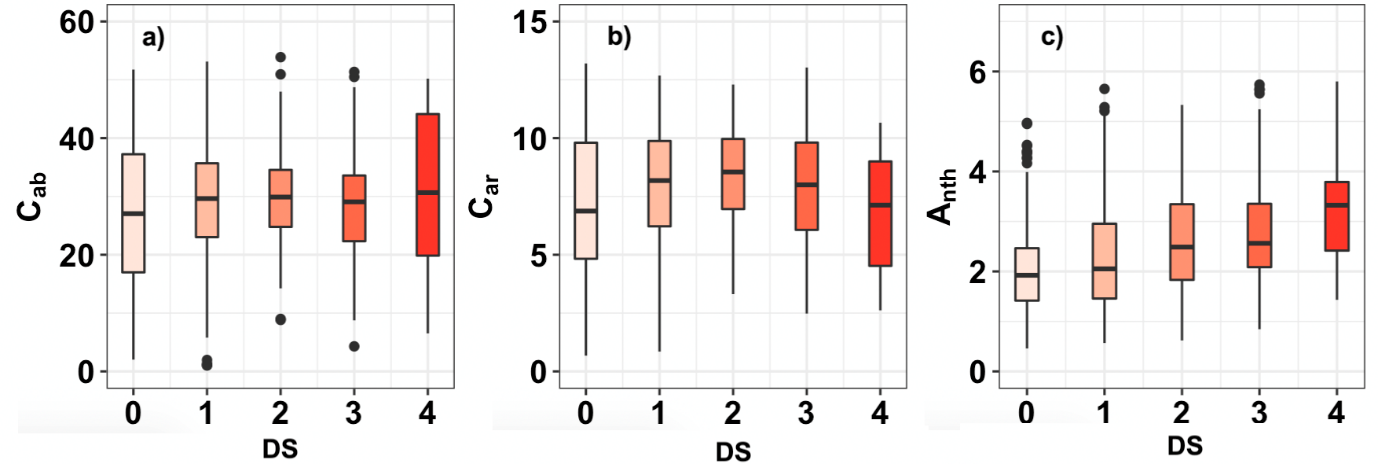


**Fig. S7.** Boxplots of inverted chlorophyll (in µg/cm^2^), carotenoid (b; in µg/cm^2^) and anthocyanin content (c; in µg/cm^2^) as a function of the tree-based disease severity (DS) rating scale (0-4). For the analysis, we used 1,426 almond trees grouped by DS class: where DS_0_ = 657 trees, DS_1_ = 359 trees, DS_2_ = 214 trees, DS_3_ = 142 trees and DS_4_ = 54 trees).


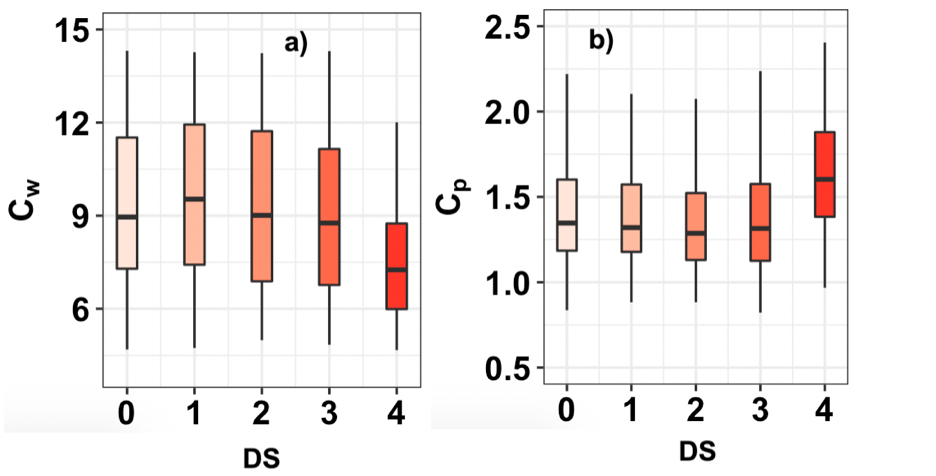


**Fig. S8**. Boxplots of inverted leaf equivalent water thickness (a; Cw in mg/cm^2^) and leaf protein content (b; C_p_ in mg/cm^2^) as a function of the tree-based disease severity (DS) rating scale (0-4). For the analysis, we used 1,426 almond trees grouped by DS class: where DS_0_ = 657 trees, DS_1_ = 359 trees, DS_2_ = 214 trees, DS_3_ = 142 trees and DS_4_ = 54 trees).


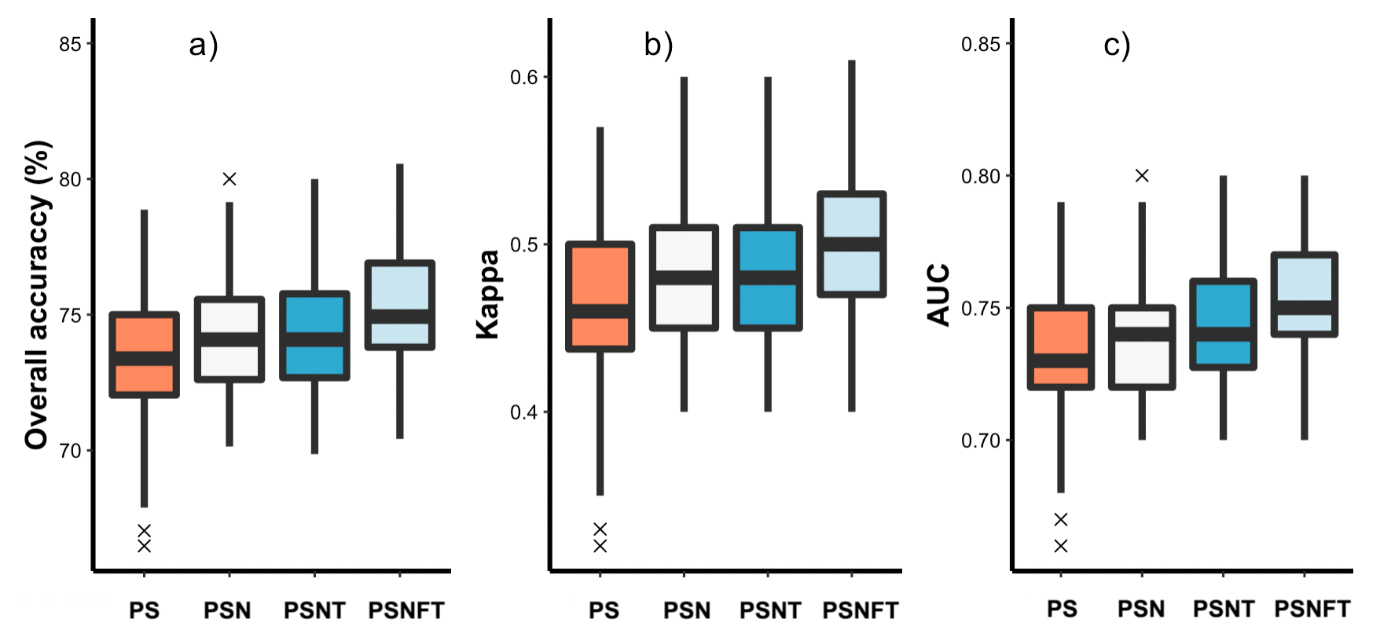


**Fig S9.** Overall accuracy (a), kappa (b) and area under the curve (AUC; c) for the studied RS-based SVM models (PS, PSN, PSNFT) and PSN + crown temperature (T_c_). Where the PS model included the VIF-Wilks’ lambda indices in the VNIR and plant traits retrieved by inversion (C_ab_, A_nth_, C_ar_, C_w_, LAI, and LIDF_a_); PSN model added leaf protein content and nitrogen indices to PS model; PSNT added the T_c_ to PSN model and PSNFT model also included the chlorophyll fluorescence emission.


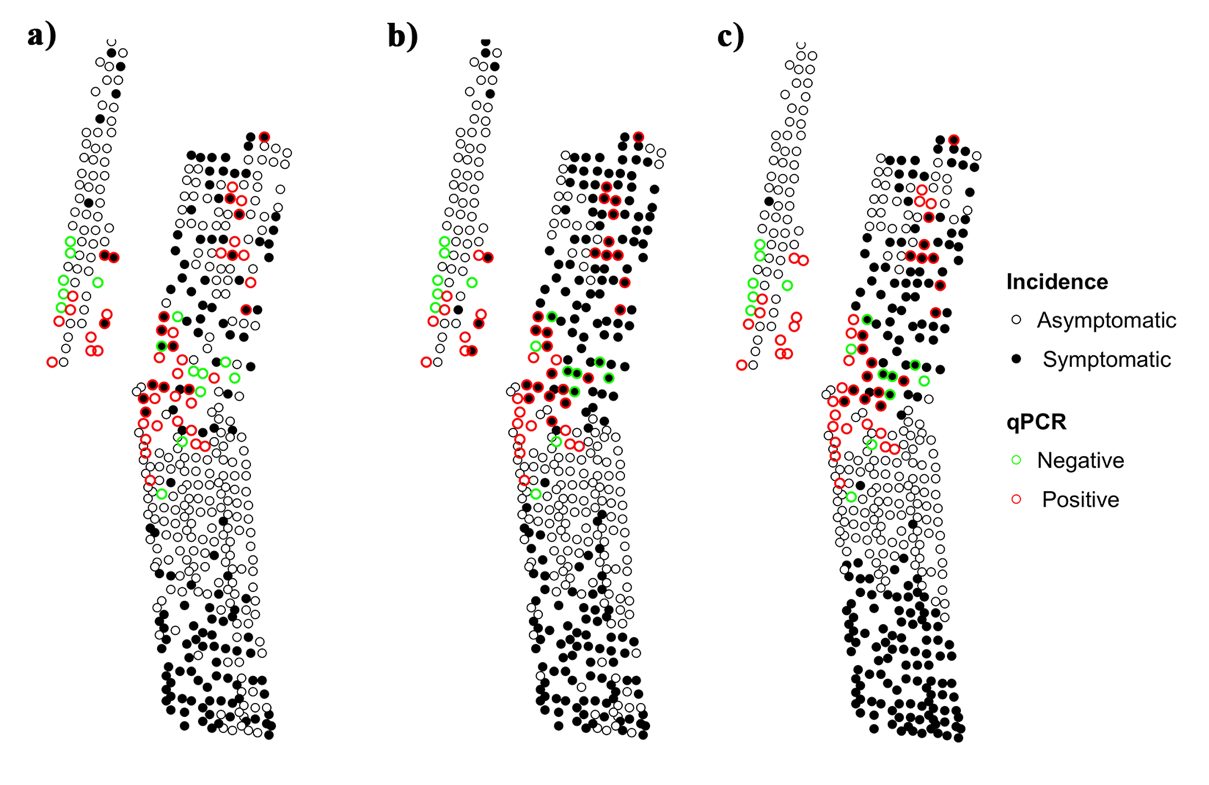


**Fig. S10.** Location of almond trees in orchard 4 affected by leaf scorch caused by *Xylella fastidiosa* (*Xf*) according to their disease status (asymptomatic vs. *Xf*-symptomatic after visual inspection) and *Xf*-infection status determined by qPCR test (a), predicted by the PSNFT model (b) and by PS (c) coupled with the stochastic spread model using a 5% sampling size, respectively.

**References**

Bao Y.; Xu K.; Min J.; Xu J.,  Estimating wheat shoot nitrogen content at vegetative stage from in situ hyperspectral measurements. Crop Sci. 53. (2013) 2063–2071. 10.2135/cropsci2013.01.0012.

Datt B.,  Remote sensing of chlorophyll a, chlorophyll b, chlorophyll a+b, and Total carotenoid content in Eucalyptus leaves. Remote Sens. Environ. 66. (1998) 111–121. 10.1016/S0034-4257(98)00046-7.

Hernández-Clemente R.; Navarro-Cerrillo R.M.; Suárez L.; Morales F.; Zarco-Tejada P.J.,  Assessing structural effects on PRI for stress detection in conifer forests. Remote Sens. Environ. 115. (2011) 2360–2375. 10.1016/j.rse.2011.04.036.

Hernández-Clemente R.; Navarro-Cerrillo R.M.; Zarco-Tejada P.J.,  Carotenoid content estimation in a heterogeneous conifer forest using narrow-band indices and PROSPECT+DART simulations. Remote Sens. Environ. 127. (2012) 298–315. 10.1016/j.rse.2012.09.014.

Roujean J.L.; Breon F.M.,  Estimating PAR absorbed by vegetation from bidirectional reflectance measurements. Remote Sens. Environ. 51. (1995) 375–384. 10.1016/0034-4257(94)00114-3.

Vogelmann J.E.; Rock B.N.; Moss D.M.,  Red edge spectral measurements from sugar maple leaves. Int. J. Remote Sens. 14. (1993) 1563–1575. 10.1080/01431169308953986.

Zarco-Tejada P.J.; Miller J.R.; Mohammed G.H.; Noland T.L.,  Chlorophyll fluorescence effects on vegetation apparent reflectance: I. Leaf-level measurements and model simulation. Remote Sens. Environ. 74. (2000) 582–595. 10.1016/S0034-4257(00)00148-6.
